# Using CRISPR barcoding as a molecular clock to capture dynamic processes at single-cell resolution

**DOI:** 10.1101/gr.280915.125

**Published:** 2026-05

**Authors:** Yolanda Andres-Lopez, Alice Santambrogio, Ioannis Kafetzopoulos, Christopher D. Todd, Carla El Khouri-Gonzalez, J-Elliot Gonzalez-Alvarez, Celia Alda-Catalinas, Stephen J. Clark, Wolf Reik, Irene Hernando-Herraez

**Affiliations:** 1 Instituto de Biología Molecular de Barcelona, Consejo Superior de Investigaciones Científicas (IBMB-CSIC), 08028 Barcelona, Spain;; 2 PhD program in Bioinformatics, University of Barcelona, 08028 Barcelona, Spain;; 3 Epigenetics Programme, Babraham Institute, Cambridge CB22 3AT, United Kingdom;; 4 Altos Labs, Granta Park, Cambridge CB21 6GQ, United Kingdom;; 5 Research and Development, GSK, Stevenage SG1 2NFX, United Kingdom

## Abstract

Biological processes are inherently dynamic, yet current methods for capturing temporal changes remain limited. Here, we present scDynaBar, a novel approach that combines CRISPR-Cas9 dynamic barcoding with single-cell sequencing. In this system, genetic barcodes gradually accumulate mutations over time; these barcodes are sequenced alongside the transcriptome of individual cells. We propose that the divergence of these barcodes from the original sequence can serve as a record of the timing of cellular events. To demonstrate the potential of this method, we track the transition from a pluripotent state to a two-cell (2C)-like state in mouse embryonic stem cells (mESCs), providing evidence for the transient nature of the 2C-like state. Additionally, our system shows consistent mutation rates across diverse cell types in a mouse gastruloid model, highlighting its applicability to other biological systems. This approach not only improves our ability to study single-cell dynamics but also opens up new possibilities for recording other temporal signals—in other words, using dynamic barcoding as a molecular clock in individual cells.

Cellular behavior is intrinsically dynamic, undergoing alterations in response to internal and external stimuli. Different methodologies are commonly used to track or infer these changes. However, these methods present several limitations. For example, methods based on live-cell fluorescence microscopy are often incompatible with in vivo studies and are restricted in terms of long-term and throughput capabilities (Maška et al. 2023). Other methods, such as those based on inference algorithms using single-cell RNA sequencing (scRNA-seq) have low temporal resolution, as scRNA-seq only captures a snapshot of gene expression at one specific time point (Haghverdi et al. 2016; La Manno et al. 2018).

DNA memory systems based on CRISPR technology have emerged as valuable tools for recording dynamic events (Sheth and Wang 2018; Li et al. 2019; Pickar-Oliver and Gersbach 2019). These systems exploit CRISPR-based techniques to induce permanent genetic modifications at precise genomic locations. By temporally regulating the delivery of CRISPR components, these systems can generate DNA mutations that function as molecular barcodes, enabling retrospective analysis of cellular activities. A variety of DNA memory devices have been developed to track a wide range of biological signals (Sheth and Wang 2018), including gene expression (Schmidt et al. 2018; Bhattarai-Kline et al. 2022), chemical exposure (Sheth et al. 2017; Farzadfard and Lu 2018; Schmidt et al. 2018), lineage of origin (McKenna et al. 2016; Kalhor et al. 2018; Loveless et al. 2021), clonality (Zhang et al. 2021; Choi et al. 2022), activation of signaling pathways (Tang and Liu 2018), and inflammatory responses (Perli et al. 2016; Park et al. 2021). Furthermore, several methods now enable single-cell resolution readouts. These approaches typically employ guide RNAs (gRNAs) targeting polyadenylation (poly(A)) sites, allowing simultaneous sequencing of both single-cell transcriptomes and DNA barcodes (McKenna and Gagnon 2019; Wagner and Klein 2020). This integrated strategy has been successfully applied to track lineage of origin in developmental (Frieda et al. 2017; Alemany et al. 2018; Raj et al. 2018; Spanjaard et al. 2018; Chan et al. 2019; Bowling et al. 2020; Li et al. 2023) and cancer studies (Quinn et al. 2021; Simeonov et al. 2021; Yang et al. 2022). More recently, prime editing has also been applied to HEK293T cells to track their lineage of origin at the single cell level (Choi et al. 2022).

Dynamic CRISPR barcoding has also been proposed as a strategy for encoding temporal information (Park et al. 2021). This approach relies on self-targeting gRNAs, a variant of the canonical form of CRISPR-Cas9 gRNAs with the unique ability to repeatedly target their own sequence (Perli et al. 2016; Kalhor et al. 2018). The continuous mutagenesis leads to a gradual accumulation of mutations over time, which can be interpreted as a molecular record of elapsed time (Park et al. 2021). However, because the target site is located within the gRNA locus itself, which lacks polyadenylation, this approach is incompatible with standard scRNA-seq techniques, limiting its use in single-cell studies. Here, we aimed to develop a system that (1) gradually accumulates mutations over extended periods and (2) allows simultaneous profiling of genetic barcodes and gene expression at the single-cell level, using standard sequencing technologies for easy implementation. This approach establishes a framework to track time-resolved processes in single cells.

## Results

### Dynamic barcoding

**Figure 1.**
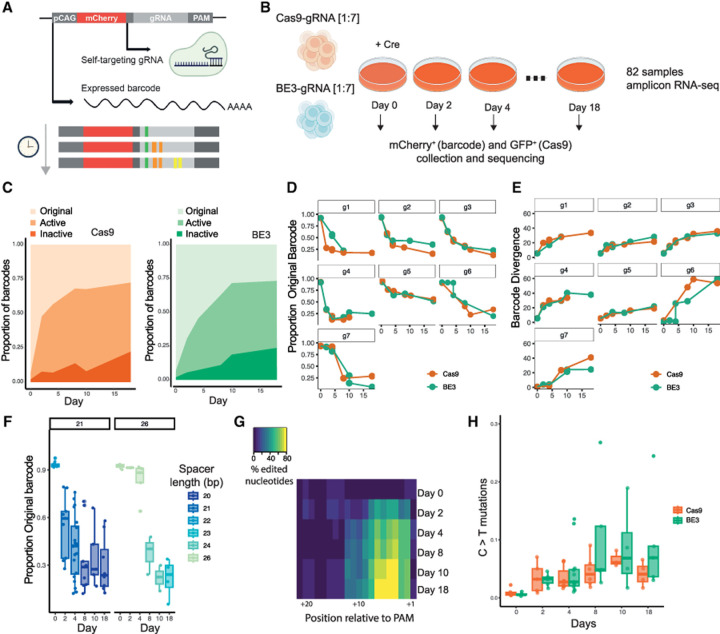
Dynamic barcoding. (*A*) Schematic representation of dynamic barcoding with a poly(A) readout. (*B*) Schematic representation of the experimental setup. Double-positive cells were defined as GFP^+^ (Cas9) and mCherry^+^ (Barcode) cells and were collected by FACS at all sequenced time points (days 0, 2, 4, 8, 10, and 18). In total, 82 replicate-1 libraries with coverage greater than 200 reads were analyzed (40 Cas9 and 42 BE3; see setion Bulk Barcode Analysis in the Methods). (*C*) Proportion of barcodes based on their mutational profiles for Cas9 (*left*) and BE3 (*right*). Barcodes are grouped into three categories: edits on the protospacer with intact PAM motif (active), absence of PAM motif (inactive), and uncut gRNA (original). Data represent the average across all seven gRNAs. (*D*) Proportion of original barcodes over time across different gRNAs. Each point corresponds to an individual sample. (*E*) Barcode mean divergence from the original barcode sequence over time, considering mismatches, gaps, and gap extensions across different gRNAs (on average, 449 different barcode sequences were detected per sample). Each point corresponds to an individual sample. (*F*) Proportion of original barcodes over time for gRNAs with 21 bp spacers (*left*) or 26 bp spacers (*right*). Box plots are colored by the mean spacer length at different time points (Cas9 system). Sample size (*left* to *right*): for 21 bp spacers, n = 10, 9, 20, 9, 5, and 8; for 26 bp spacers, n = 4, 2, 5, 3, 3, and 4. (*G*) Mean percentage of the original nucleotide over time, aligned relative to the PAM sequence in the Cas9 system (n = 39). (*H*) Fraction of C > T mutations over time, considering all different gRNAs classified by Cas9 versions. Sample size (*left* to *right*): n = 7, 7, 5, 6, 11, 14, 7, 5, 4, 4, 6, and 6. For all box plots, the boxes represent the interquartile range (IQR), with the horizontal line *inside* each box indicating the median.

To enable dynamic barcoding and a single-cell readout, we engineered self-targeting gRNAs (Perli et al. 2016; Kalhor et al. 2018) to be retrieved from a polyadenylated transcript (expressed barcode). We also included an mCherry reporter, which enables easy visualization and selection of targeted cells (Fig. 1A).

To investigate the dynamics of the system and to test its ability to detect the barcodes at the RNA level, we performed a time course experiment using seven different gRNAs with varying protospacer sizes (21 or 26 bp) and nucleotide compositions (Supplemental Fig. S1). We also aimed to evaluate the relative efficacies of barcoding using standard Cas9 and a base editing system, as the latter avoids inducing double-strand breaks. Specifically, we used the BE3 base editor, which consists of a Cas9 nickase fused to the cytidine deaminase APOBEC and the uracil DNA glycosylase inhibitor (UGI) domain (Komor et al. 2016). The UGI domain facilitates uracil retention, ensuring precise C:G to T:A substitutions within a 4 to 8 nucleotide (nt) window (Komor et al. 2016). In its absence, the frequency of indels increases, along with a higher incidence of other substitutions (Komor et al. 2016; Wang et al. 2017). Therefore, to enhance mutational diversity in our approach, we used a BE3 system lacking this domain (APOBEC–XTEN–dCas9(A840H)) (Komor et al. 2016). Both Cas9 constructs contained a constitutive promoter (CMV early enhancer fused to modified chicken beta actin promoter) and a GFP reporter. Expression of the constructs was controlled by a Cre-responsive FLEX switch, ensuring that expression occurred only after recombination. We cotransfected individual gRNAs together with one of the two Cas9 constructs into mouse embryonic stem cells (mESCs). After stable integration, we activated Cas9 by adding Cre recombinase to the culture media and then collected cells from this mixed polyclonal population at various time points, up to day 18 (Fig. 1B). We then selected cells positive for both reporters (GFP and mCherry) by fluorescence-activated cell sorting (FACS) and subsequently performed bulk amplicon sequencing on their RNA (Methods) (Fig. 1B; see Supplemental Fig. S2).

To investigate the general dynamics of the systems, we first classified the barcode sequences into three groups: (1) the original barcode, in which no editing occurred; (2) an active barcode, in which editing occurred in the protospacer region but the PAM sequence remained uncut; and (3) an inactive barcode, in which editing occurred in both the protospacer region and the PAM sequence, preventing further mutagenesis. Our data are consistent with sequential editing, as they show increased level of active and inactive barcodes, as well as a decreased frequency of original barcodes, over time (Fig. 1C). The increased editing rate is consistent across all seven gRNAs tested, as well as with both versions of Cas9 (Jonckheere–Terpstra test, *P* = 2 × 10^−13^) (Fig. 1D). Background editing in the absence of Cre (at day 0) was low (averaging 3% across experiments); this could result from spontaneous Cas9 expression (Supplemental Fig. S3). We next assessed the divergence of the edited barcodes by comparing their sequences to the original barcode. In this analysis, we performed pairwise alignments of more than 100,000 barcode sequences and calculated a divergence score considering mismatches, gaps, and gap extensions. Similarly, all seven gRNAs showed a sequential increase in mean divergence over the 18 days (Jonckheere–Terpstra test, *P* = 8 × 10^−16^), providing strong evidence for continuous mutagenesis and their capacity to record molecular events (Fig. 1E; Supplemental Fig. S4). In agreement with previous studies, we also observed that in the Cas9 system, 26 bp gRNAs exhibit a slower editing rate compared with 21 bp gRNA during the initial 4 days (day 4, Mann–Whitney–Wilcoxon test, *P* = 0.0005) (Fig. 1F; Kalhor et al. 2017). After this time point, the frequency of intact barcodes for both the 21 bp and the 26 bp gRNAs became very similar. This could be attributed to the accumulation of deletions in the 26 bp gRNAs (Fig. 1F), potentially leading to an increased editing rate at later time points.

Next, we analyzed the distribution of mutagenesis relative to the PAM of each gRNA in the Cas9 system. As expected from the activity of Cas9, we observed a preferential accumulation of mutations near the cut site, particularly within 3 nt upstream of the PAM site (Fig. 1G). We then compared the specific types of edits in the BE3 system to those in the standard Cas9. Notably, we observed a similar accumulation of indels over time in both systems (Mann–Whitney–Wilcoxon test, *P* = 0.45) (Supplemental Fig. S5). This could be attributed to the absence of the UGI domain, which may lead to DNA repair via alternative cellular mechanisms, ultimately resulting in indel formation. Despite the absence of the UGI domain, our BE3 system exhibited a slightly higher frequency of C > T mutations at most time points, with an average difference of 6.9% (Fig. 1H).

### Reproducibility and window of activity

**Figure 2.**
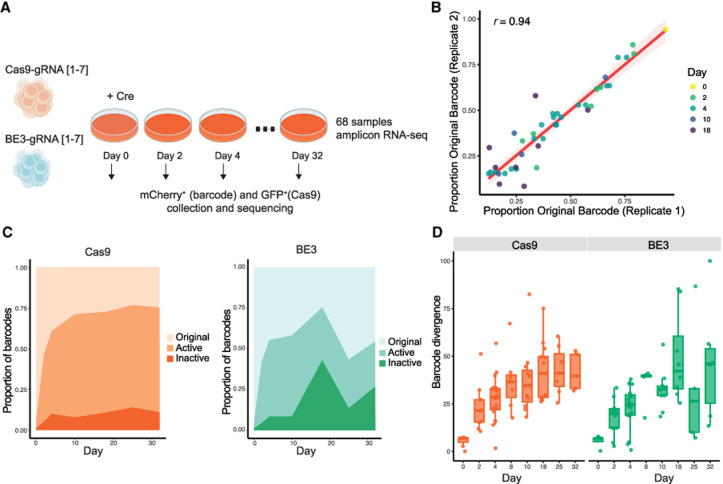
Reproducibility of barcode dynamics between two different replicates and window of activity. (*A*) Schematic representation of the experimental setup. Double-positive cells were defined as GFP^+^ (Cas9) and mCherry^+^ (Barcode) cells and were collected by FACS at all sequenced time points (days 0, 2, 4, 8, 10, 18, 25, and 32). In total, 68 replicate-2 libraries with coverage of more than 200 reads were analyzed (39 Cas9 and 29 BE3; see section “Bulk barcode analysis” in the Methods. (*B*) Scatter plot of original barcode frequencies in independent biological replicates. Each point represents one biological condition (defined by gRNA, experiment type, and time point), with replicate 1 on the *x*-axis and replicate 2 on the *y*-axis. Points are colored by day. A total of 86 samples were analyzed, including 50 from the Cas9 experiment and 36 from the BE3 experiment. The Pearson correlation coefficient between replicates is *r* = 0.94. (*C*) Proportion of barcodes based on their mutational profile: edits on the protospacer with intact PAM motif (active), lack of PAM motif (inactive), and uncut gRNA (original) according to Cas9 (*left*) or BE3 (*right*). Data represent the average across all seven gRNAs (Cas9 n = 39; BE3 n = 29). (*D*) Barcode divergence from the original barcode sequence over time, considering mismatches, gaps, and gap extensions, pooling all gRNA for Cas9 (*left*) and BE3 (*right*). Sample size (*left* to *right*): for Cas9, n = 7, 10, 19, 7, 11, 7, 13, and 5; for BE3, n = 8, 10, 19, 5, 8, 5, and 6. For all box plots, the boxes represent the interquartile range (IQR), with the horizontal line *inside* each box indicating the median.

We next aimed to extend the duration of mutagenesis to determine the maximum period during which the system can remain active and continue to generate mutations in an independent biological replicate. In this experiment, each of the seven gRNAs was individually cotransfected with one of the Cas9 constructs and then processed at various time points, up to day 32 (Fig. 2A). First, to assess consistency across experiments, we compared the percentage of original barcodes between replicates for conditions in both biological replicates (considering gRNA, day, and experiment; n = 86). We observed consistent results between biological replicates, with a Pearson's correlation coefficient of 0.94 (*P* < 2.2 × 10^−16^), indicating the high reproducibility of our system (Fig. 2B; Supplemental Fig. S6). We then quantified the percentage of barcodes that appeared in both replicates, defined as the mean of the intersections divided by the union of barcodes. This analysis revealed that ∼43% of barcodes were shared, and this fraction remained stable across time points (Supplemental Fig. S7). These findings suggest that mutation patterns are not entirely random and that the same mutations tend to arise independently across replicates, likely reflecting biases in DNA repair mechanisms.

Focusing on replicate 2, we observed that the Cas9 system remained active until day 25, albeit working at a slower rate (Fig. 2C,D). In contrast, the BE3 system exhibited a significant increase in the frequency of original barcodes and a decrease in sequence divergence at day 25 (Fig. 2C,D). Consistent with this, after accounting for sample coverage, the number of unique barcodes tended to increase in the Cas9 system up to day 18, whereas in the BE3 system, this increase occurred only until day 4 (Supplemental Fig. S8). We hypothesize that this may result from differences in cellular fitness. Nonetheless, future experiments are needed to better understand this phenomenon.

In conclusion, we demonstrate that both systems are effective for sequential barcoding, with BE3 achieving comparable levels of barcode divergence in experiments conducted over 2 weeks and with Cas9 being more suitable for long-term applications.

### Simultaneous detection of genetic barcodes in single cells

**Figure 3.**
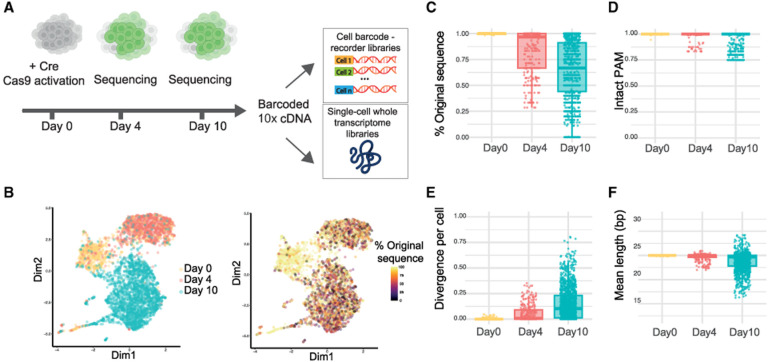
Single-cell readout. (*A*) Schematic representation of the experimental setup. Double-positive cells were collected at days 0, 4, and 10 after Cre induction and processed using the 10x Genomics platform. Barcoded cDNA was used to prepare two different libraries: (1) a targeted amplicon library enriched for the scDynaBar cassette (“cell barcode–recorder library”) and (2) a whole-transcriptome gene expression library. (*B*) t-SNE plot colored by cluster: noninduced (day 0) and induced (day 4 or day 10; *left*). Color indicates the mean percentage of the original barcode sequence per cell (*right*). (*C*–*F*) Box plot of results at days 0 (n = 495), 4 (n = 626), and 10 (n = 1117) showing the percentage of the original barcode sequences per cell (*C*), the mean proportion of barcodes with full PAM sequence per cell (*D*), mean barcode divergence from the original barcode sequence per cell (*E*), and mean length of the barcodes per cell (*F*). For all box plots, the boxes represent the IQR, with the horizontal line *inside* each box indicating the median. Each dot represents an individual cell, with data points displayed within the 95% confidence interval.

We then evaluated whether genetic barcodes could be simultaneously recovered with transcriptomic data using standard scRNA-seq. First, based on our previous observations, we selected gRNA g3 in combination with the canonical form of Cas9. We then established a clonal cell line to minimize potential variability owing to differences in integration sites among individual cells. Using qPCR on genomic DNA, we determined that this cell line likely contains a single integration (Methods) (Supplemental Fig. S9). We grew these cells under serum/LIF conditions, and following Cre induction, we FACS-sorted double-positive cells (for GFP and mCherry) on days 4 and 10 (Fig. 3A). We also included a noninduced group that showed minimal Cas9-GFP expression at day 10 (GFP = 0.1%), confirming low background activity.

Cells were processed using the 3′ 10x Genomics platform, in which they were encapsulated in droplets with barcoded Poly(dT)-coated beads and subjected to whole-transcriptome amplification following the standard 10x Genomics scRNA-seq 3′ protocol. We then specifically amplified and sequenced the genetic barcodes from the cell-barcoded cDNA (Fig. 3A). Overall, we successfully recovered genetic barcodes from 97% of cells that passed transcriptome quality control, demonstrating the efficiency of our approach for simultaneous transcriptome and barcode detection.

t-Distributed stochastic neighbor embedding (t-SNE) analysis on the transcriptomes revealed three clusters of cells, corresponding to the different time points: noninduced (day 0; *n* = 495), induced at day 4 (*n* = 626), and induced at day 10 (*n* = 1117) (Fig. 3B). These clusters likely reflect batch effects rather than biological differences, as only 64 genes were differentially expressed across all comparisons, with no consistent changes except for the Cas9 gene.

For each cell, we assessed barcoding levels by analyzing the following: the proportion of reads carrying the original barcode sequence, the mean barcode divergence score (calculated as described previously), the average length of the barcode sequence, and the percentage of barcodes carrying a PAM sequence (Fig. 3C–F). Our findings align with the results in bulk, showing progressive editing over time (Jonckheere–Terpstra test, *P* = 2 × 10^–4^, 1000 permutations). We observed a decrease in the presence of original sequences along with a decline of the proportion of barcodes carrying a PAM motif (Fig. 3C,D). Across cells, the mean ± SD percentage of original sequences was 100.0 ± 0.0% at day 0, 81.0 ± 23.5% at day 4, and 64.4 ± 28.2% at day 10. The most frequent barcode sequences are listed in Supplemental Table S1, illustrating the progressive increase in edited barcodes over time. Additionally, we observed an increase in barcode divergence and greater variability in their lengths (Fig. 3E,F). We also detected an increased number of unique barcode sequences per cell (Supplemental Fig. S10A), which likely represent sequence derivatives of one another. To test this, we quantified barcode similarity as pairwise distance within and across cells. Using 1000 permutations on groups of 15 cells, we found that barcodes from the same cell were significantly more similar than those from different cells (*P* < 2.2 × 10^–16^, Wilcoxon rank-sum test), supporting a model of progressive, accumulative editing over time (Supplemental Fig. S10B). Finally, we observed that the 1480 edited gRNA sequences were unlikely to have off-target effects that could affect cell viability (Supplemental Table S2). Altogether, these results demonstrate the effectiveness of dynamic barcoding in single cells and its capability to simultaneously capture barcodes and transcriptomic information using standard single-cell technologies.

### Recording dynamic transitions

**Figure 4.**
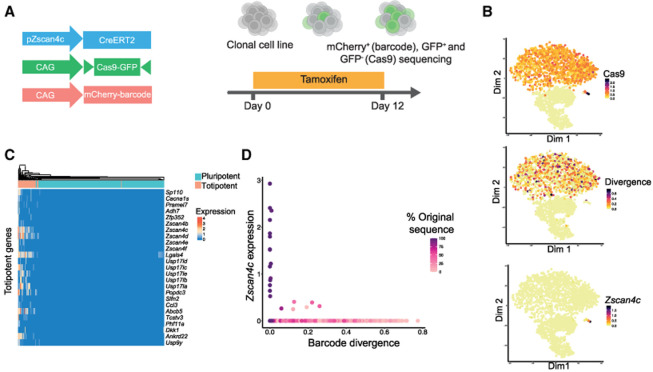
Single-cell recording of 2C-like transitions with scDynaBar. (*A*) Schematic representation of the experimental setup for tracking 2C-like state transitions. Double-positive cells (∼73%) and a subset of GFP-negative cells (∼27%) were collected for sequencing into a single 10x Genomics run at day 12. (*B*) t-SNE plot colored by Cas9 expression (*top*), mean barcode divergence from the original barcode sequence per cell (*middle*), and *Zscan4c* expression (log_2_ normalized counts; *bottom*). (*C*) Heatmap displaying gene expression (log_2_ normalized) for top markers for the totipotent cluster. (*D*) Scatter plot displaying *Zscan4c* expression (log_2_ normalized; *y*-axis) against barcode divergence (*x*-axis) per cell, with color indicating the percentage of original barcode sequences.

Having established scDynaBar in single cells, we next applied it to track transitions between pluripotency and totipotency-like states. Under standard serum/LIF culture conditions, a small population (<1%) of mESCs spontaneously emerges that exhibits features resembling the totipotent 2C stage (Macfarlan et al. 2012; Li and Izpisua Belmonte 2018). These features include a specific transcriptional profile, such as the expression of *Zscan4* genes, increased histone mobility, DNA demethylation, and the capacity to contribute to both embryonic and extraembryonic tissues (Macfarlan et al. 2012; Bošković et al. 2014; Eckersley-Maslin et al. 2016). Although it is believed that the 2C-like state cannot self-propagate but rather continuously undergoes transitions to a pluripotent state, these dynamics have not been directly measured (Macfarlan et al. 2012; Rodriguez-Terrones et al. 2018). To track 2C-like conversions, we integrated our barcoding approach with a *pZscan4*-CreERT2 construct (Fig. 4A; Zalzman et al. 2010). With this setup, cells entering the 2C-like state permanently activate the barcoding cassette upon tamoxifen treatment. After establishing a monoclonal line, cells were treated with tamoxifen for 12 days. Following treatment, we observed an increase in GFP-positive cells (9% tamoxifen-treated cells; 0.1% in untreated cells). Double-positive cells (GFP- and mCherry-positive) were FACS-sorted and processed for single-cell RNA-seq and barcoding sequencing (Fig. 4A). We also sequenced a portion of GFP-negative cells as a control.

After cell and barcode quality control, we recovered 3352 GFP-positive cells and 1270 Cas9-GFP-negative cells (with a 98% barcode recovery rate). t-SNE analysis identified three clusters corresponding to Cas9-GFP-negative cells and two clusters representing Cas9-GFP-positive cells. Within the Cas9-GFP-positive population, we observed variable levels of barcode divergence, consistent with the activation of barcoding at distinct time points during our time course (Fig. 4B). t-SNE analysis also revealed a cluster of 24 cells corresponding to 2C-like cells, characterized by expression of totipotency-associated markers, such as *Zscan4c* (Fig. 4B,C).

Cells in the pluripotent state, characterized by a lack of *Zscan4c* expression, exhibited high variability in their barcode divergence scores (divergence Brown–Forsythe test, *P* = 0.01; original barcode sequence Brown–Forsythe test, *P* = 0.0006) (Fig. 4B). This high variability indicates that the CRISPR barcoding system was activated earlier upon entry into the 2C-like state at different time points in each of the cells. As a result, the continuous activity of the barcoding system led to different levels of barcode divergence over time. In contrast, all cells in the 2C-like state (i.e., with *Zscan4c* expression) displayed low barcode divergence, along with a high proportion of the original barcode sequence (divergence Mann–Whitney–Wilcoxon test, *P* = 0.0007; original barcode sequence Mann–Whitney–Wilcoxon test, *P* = 0.0004) (Fig. 4D). This low divergence cannot be attributed to reduced coverage of the barcode cassette in this population or other technical factors (Supplemental Fig. S11). The absence of 2C-like cells with high barcode divergence indicates that they transitioned into this state only recently. These findings support the prevailing hypothesis in the field that embryonic stem cells (ESCs) undergo continuous dynamic transitions into and out of the 2C-like state, a phenomenon that has previously been difficult to capture directly (Macfarlan et al. 2012; Rodriguez-Terrones et al. 2018; Fu et al. 2020). Our results also indicate that the 2C-like cells that became pluripotent did not re-enter into the 2C-like state during the experiment. This could be attributed to a combination of factors: probability, as only ∼1% of the cells are in the 2C-like state at any given time, and potential inhibitory or promoting signals that might regulate re-entry into this state. To further explore these dynamics, we developed a stochastic simulation model of transitions between pluripotent and 2C-like states (see Methods). The simulations recapitulate our experimental observations, indicating that the 2C-like state is transient, with an average duration of 1.55 days, and that re-entry into this state after a previous activation is rare (∼0.3%) in the observed period (Supplemental Fig. S12).

To identify candidate genes that may regulate these transitions, we performed a correlation analysis between the transcriptome profiles and barcode divergence scores in pluripotent cells. *Dppa4*, a gene previously shown to play a role in the 2C-like state, emerged as one of the top correlated genes. (Supplemental Table S3; Supplemental Fig. S13). Specifically, pluripotent cells with higher barcode divergence exhibit higher *Dppa4* expression, whereas pluripotent cells with lower divergence have lower *Dppa4* levels, and 2C-like cells show the lowest levels.

These observations suggest that *Dppa4* expression gradually increases after cells exit the 2C-like, potentially contributing to pluripotency stabilization and to cells priming for future 2C-like transitions. Consistently, previous studies have demonstrated that *Dppa4* depletion inhibits the 2C-like reprogramming in mESCs (De Iaco et al. 2019; Eckersley-Maslin et al. 2019), supporting the role for *Dppa4* in gradually preparing pluripotent cells for future 2C-like transitions. Other genes correlated with the divergence score that may also be involved in the 2C-like transition include jumonji domain containing 8 (*Jmjd8*) or zinc finger protein 61 (*Zfp61)* (Supplemental Fig. S13).

These results show the capability of scDynaBar in capturing temporal dynamic transitions between cellular states, providing an effective approach for investigating behaviors that were previously difficult to measure directly.

### Dynamic barcoding in mouse gastruloids

**Figure 5.**
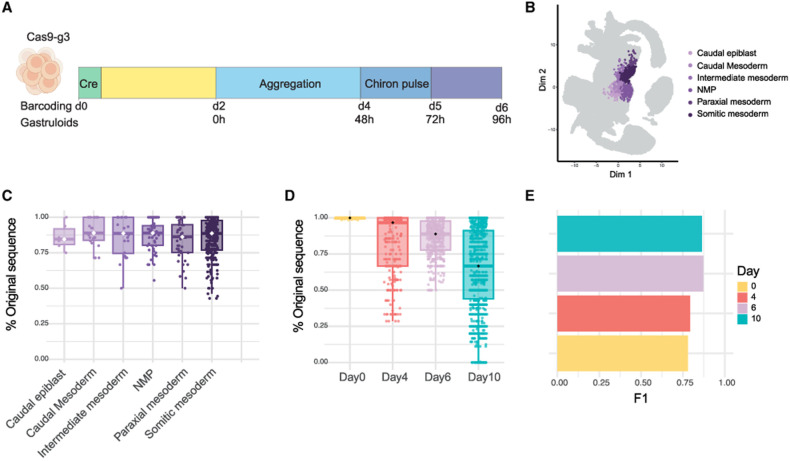
Barcoding rate in gastruloids and temporal prediction. (*A*) Schematic of the experimental design for gastruloid formation and barcoding. Cre induction was performed on day 0 (d0). After 2 days (yellow bar), cells were plated for aggregation (light blue). On day 4 (d4), a Chiron pulse (CHIR99021) was applied to promote gastruloid elongation and cell differentiation (dark blue). Gastruloids were further cultured (purple bar) and collected on day 6 (d6) of barcoding (96 h of gastruloid culture) for single-cell sequencing (18 gastruloids). (*B*) UMAP projection of a reference atlas of mouse embryos (E6.5 and E8.5). Nearest neighbor analysis was used to assign cell-type labels to the gastruloid cells based on their proximity to the cells in the reference atlas. Gastruloid cells are overlaid in purple, colored by lineage. (*C*) Box plot showing the percentage of the original barcode sequence per cell type in day-6 gastruloids. (*D*) Box plot showing the distribution of the proportion of original barcode sequences across different time points (days 0, 4, 6, and 10) in single cells. Each point represents one cell. (*E*) Bar plot showing the F1 score for each time point (days 0, 4, 6, and 10) obtained from the random forest model on the test data. Bars are colored according to the corresponding day. For all box plots, the boxes represent the IQR with the horizontal line *inside* each box indicating the median.

To investigate the mutation rate across other cell types, we applied scDynaBar to mouse gastruloids, which are three-dimensional structures that mimic early developmental processes and contain a diverse array of cell types (Beccari et al. 2018). For this, we used our previously established mESC monoclonal cell line (Fig. 3). Two days after Cre induction, mESCs were seeded for aggregation and subsequent gastruloid formation (Fig. 5A; Beccari et al. 2018). Six days after induction, day 4 gastruloids were dissociated, and the cells were processed for single-cell RNA and barcode sequencing. These gastruloids exhibited normal morphology and low levels of cell death (Supplemental Fig. S14). After the cell and barcode quality controls, we recovered 1057 Cas9-GFP-positive cells (i.e., GFP counts greater than one) and assigned cell types by mapping RNA expression profiles to a reference atlas of mouse embryos, from E6.5 to E8.5 (Fig. 5B; Pijuan-Sala et al. 2019). This analysis revealed a bias toward the mesodermal lineage, consistent with our previous studies showing that gastruloids often favor mesodermal or ectodermal cell types (Rosen et al. 2022). We then computed the proportion of the original barcode sequence and the barcode divergence score for each cell and found no significant differences between cell populations (Fig. 5C; Supplemental Fig. S15A,B), indicating that the barcoding rate is similar across these cell types (Kruskal–Wallis test, *P* = 0.3557). Comparing these data with our previous single cell data set (Fig. 3), we observed that gastruloids collected at day 6 exhibited levels of barcoding that ranged between those observed at day 4 and day 10 (Fig. 5D; Supplemental Fig. S15C). Specifically, the median value of the original barcode sequencing was 85% on day 6 compared with 96% on day 4 and 66% on day 10, consisting with a cumulative increase in mutations over time (Jonckheere–Terpstra test, *P* = 2 × 10^–4^, 1000 permutations) (Fig. 5D). We hypothesized that the gradual increase in barcode editing could be used to determine the duration of barcode activity in single cells. To test this hypothesis, we developed a temporal predictor using a random forest classifier on our single cell data sets (days 0, 4, 6, and 10). The predictor was trained on 70% of the data, incorporating barcode features as variables (mean barcode length, minimum barcode length, maximum barcode length, mean divergence, maximum divergence, and mean number of indels). Using this predictor, we achieved an accuracy of 82.1% on the test data with consistent F1 scores across all time points (Fig. 5E).

These results suggest that DNA barcodes contain temporal information and could potentially be used to estimate the duration of barcoding in single cells. However, further studies are needed to fully assess the robustness of this approach and its applicability across different cell types and biological contexts.

## Discussion

In this study, we introduce scDynaBar, a novel approach for dynamic cellular barcoding in single cells. We accomplished this by engineering self-targeting gRNAs (Kalhor et al. 2017), enabling the simultaneous capture of both genetic barcodes and the transcriptomes of individual cells. This method requires minimal adjustments to existing commercial single-cell sequencing workflows, offering high versatility and scalability. The key advantage of this approach is its ability to encode temporal signals directly into the DNA of individual cells, creating a stable, cumulative record of dynamic cellular events over time.

We demonstrated that dynamic barcoding results in the gradual increase of genetic barcode divergence from the original sequence over 7 days (10–18 days for the Cas9 system and 4 days for the BE3 system), driven by continuous targeting, with high reproducibility. This offers an advantage over other single-cell barcoding methods, in which mutations typically occur within the first 48 h (Spanjaard et al. 2018; Bowling et al. 2020). The accumulation of mutations over time can be assessed through several measures, including (1) a reduction in the proportion of original sequences, (2) a decrease in the frequency of sequences with an intact PAM motif, (3) an increase in the divergence of spacer sequences from the original sequence, and (4) an increase in the variability of their lengths. We observed comparable rates of mutagenesis and barcode divergence using standard Cas9 and the modified cytosine base editor BE3 (Komor et al. 2016), which fuses APOBEC with nCas9. Although potential adverse effects of BE3 were observed during prolonged barcoding, requiring further investigation, its efficacy was demonstrated within a 2 week period. This system could be particularly valuable for applications that are incompatible with the generation of double-strand DNA breaks.

As a proof of concept for scDynaBar's potential applications, we demonstrated its capacity to track dynamic cell transitions. By collecting data at a single end point and using a CreERT2 protein controlled by the *Zscan4c* promoter, we tracked the transition of mESCs from the pluripotent state to the 2C-like state. In this experimental setup, the divergence of barcodes from the original sequence encodes the timing of the cell transitions to the 2C-like state. Our findings showed that all 2C-like cells had recently transitioned to this state, as evidenced by a lack of mutations in their barcodes. These results provide evidence for the transient nature of the 2C-like state and support the current hypothesis that it cannot self-perpetuate (Macfarlan et al. 2012; Rodriguez-Terrones et al. 2018; Fu et al. 2020). Additionally, these findings illustrate the potential of our approach for studying complex cellular dynamics, which are currently difficult to track with existing methodologies. We note, however, that in systems with frequent reversible transitions, scDynaBar would not distinguish cells that have remained in a state from those that exited and subsequently re-entered it.

Our observations from a mouse gastruloid model revealed normal phenotypes, indicating that cells with active barcoding preserve their differentiation potential. Additionally, we observed consistent DNA repair rates across multiple mesoderm-derived cell types, suggesting potential applications across other biological contexts. Nonetheless, further experiments are required to better understand how factors such as variations in Cas9 levels, DNA repair kinetics, proliferation rates, and DNA context influence barcode divergence. We also observed differences in the barcoding rate between bulk and single-cell experiments, likely reflecting the use of a monoclonal cell line and more stringent UMI-based filtering in the single-cell experiment. Future studies should take these factors into account. Finally, although silencing of the barcoding cassette was not observed in our system, it remains possible that different modes of Cas9 induction could lead to varying levels of activity.

Finally, we believe that scDynaBar is highly adaptable and easy to implement, making it well suited for recording a wide variety of temporal events in single cells. In principle, any cellular event that can be linked to DNA editing could be recorded, offering a versatile framework to study dynamic processes over time. By modulating Cas9 activity, scDynaBar could be tailored to track the duration or intensity of specific events, such as transcription factor activity or responses to external stimuli. This persistent molecular recording complements transcriptomic profiling and provides unique opportunities to investigate how time-resolved processes or events are integrated within heterogeneous cell populations, capturing cellular histories that were previously inaccessible.

## Methods

### Vector construction

The barcoding cassette used in this study were constructed by incorporating gBlock (IDT DNA) synthesized DNA fragments, including CAG promoter, mCherry, and scaffold, into a piggyBac backbone. Different gRNAs (spacers), obtained from Sigma-Aldrich, were cloned into this vector. The sequences of these spacers are provided in the Supplemental Figure S1. The Cas9-2A-GFP vector used in this study is based on the method of Cong et al. (2013) and was cloned into a piggyBac backbone with a one-way genetic switch (FLEX) system, which uses inverted loxP and lox2272 sites to allow Cre-dependent cassette inversion, enabling controlled, inducible expression of Cas9 (Schnütgen et al. 2003). The BE3 system, kindly provided by Alexis Komor (Komor et al. 2016), was further modified by removing the UGI and then incorporating it into a piggyBac backbone as nCas9-rAPOBEC1-2A-GFP in a one-way genetic switch (FLEX) system.

The plasmid carrying CreERT2 under the control of the *Zscan4c* promoter (*pZscan4*-CreERT2 cells), as described by Zalzman et al. (2010), was generated by VectorBuilder with a puromycin resistance marker.

### mESC culture

E14 mESCs were cultured under standard serum/LIF conditions in DMEM (Gibco 11995-040) supplemented with 15% fetal bovine serum, 1 U/mL penicillin and 1 mg/mL streptomycin (Gibco 15140-122), 0.1 mM nonessential amino acids (Gibco 11140-050), 4 mM GlutaMAX (Gibco 35050-061), 50 µM β-mercaptoethanol (Gibco 31350-010), and 103 U/mL LIF (Stem Cell Institute). Cells were maintained at 37°C in a 5% CO_2_ atmosphere on gelatinized tissue-culture plates. Media was changed daily, and cells were passaged every other day using trypsin-EDTA (Thermo Fisher Scientific 25200056).

Stable cell lines were generated through transfection using FuGENE (Promega E2311), followed by drug selection and FACS. Clonal cell lines were isolated through additional rounds of subcloning.

To determine the number of copies of the barcode construct integrated in the clonal cell line used in Figures 3 and 5, a genomic qPCR was performed. Genomic DNA was extracted from wild-type (wt) mESCs and from the clonal cell line, using the DNeasy blood and tissue extraction kit (Qiagen). The qPCR was performed using the brilliant III ultrafast SYBR green QPCR master mix in a Roche LightCycler 480 instrument II following Agilent Technologies’ guidelines of one 3 min cycle at 95°C and 40 cycles of 5 sec at 95°C and 10 sec at 60°C with the primers below. Samples were tested in triplicates, and a negative control with water was included for each primer combination. Three different DNA amounts were tested: 5 ng, 10 ng, and 20 ng. For quantification, the 2^–ΔCt^ method was used, using *Gapdh* as a reference for the relative quantification of both 2n DNA amplicons (*Actb1*, *Actb2*, and Intergenic) and the integrated barcoding cassette (barcode 1 and barcode 2) (Supplemental Table S4).

Cas9 induction was carried out using Cre recombinase (Takara 631449) in media supplemented with 6 µg/mL polybrene (Sigma-Aldrich H9268), according to the manufacturer's instructions. *pZscan4c*-CreERT2 cells were treated with 4-hydroxytamoxifen (1 µM) for 12 days (Sigma-Aldrich H7904).

### Gastruloid culture

The mESC clonal cell line containing the barcoding system was cultured under standard serum/LIF conditions. Induction was carried out as before using Cre recombinase (Takara 631449) in media supplemented with 6 µg/mL polybrene (Sigma-Aldrich H9268), according to the manufacturer's instructions. After 2 days, cells were prepared for gastruloid formation using a previously described protocol (Beccari et al. 2018). Specifically, mESCs were dissociated into single cells with trypsin-EDTA (Thermo Fisher Scientific 25300096). Cells were then washed twice with prewarmed PBS (Thermo Fisher Scientific 14190144), and the pelleted cells were then resuspended in 5 mL of N2B27 medium of a 1:1 mix of DMEM/F12 (Thermo Fisher Scientific 11320033) and Neurobasal medium (Thermo Fisher Scientific 21103049), supplemented with 0.5× N-2 supplement (Cell Therapy Systems A1370701), 0.5× B-27 supplement (Thermo Fisher Scientific 17504044), 2 mM GlutaMAX, 10 U/mL penicillin–streptomycin, and 0.1 mM 2-mercaptoethanol. Cells were then diluted to a density of 7500 cells/mL in N2B27 medium, and 40 µL of this dilution was added to each well of a U-bottom 96-well suspension culture plate (Greiner Bio-One 650185) to reach a density of 300 cells per well. The cells were left to aggregate for 48 h. After this period, 150 µL of N2B27 medium containing 3 µM CHIR99021 (Department of Biochemistry, University of Cambridge) was added. Every 24 h, 150 µL of the medium was replaced with fresh N2B27 medium without CHIR99021. On day 4 of gastruloid culture (6 days after Cre induction), gastruloids were harvested for sequencing: They were transferred to an Eppendorf tube, rinsed with PBS, and dissociated into single cells using Accutase (StemPro A1110501). Cells were then washed twice with 5 mL of PBS containing 0.04% BSA (Gibco 15260037) to remove the Accutase, followed by filtration through a 50 µm strainer (Sysmex 1050553). The cell count and viability were determined using the Countess II automated cell counter prior to single-cell sequencing.

### Bulk barcode sequencing

Bulk barcode sequencing was performed on a mixed, nonclonal population. After stable integration and induction, cells were sorted at various time points using either the BD Aria III or the BD influx high-speed cell sorter. About 8000 to 12,000 cells positive for GFP and mCherry were collected for each sample. RNA was then extracted using the RNeasy micro kit (Qiagen 74004). To amplify the gRNA loci, reverse transcription was performed as previously described (Clark et al. 2018), with 1 µL of primer (GTGACTGGAGTTCAGACGTGTGCTCTTCCGATCT-T(30)) and 500 ng of RNA. cDNA was amplified in a 20 µL reaction using KAPA HiFi HotStart ReadyMix (KAPA Biosystems KK2502) with the following primers: forward, 5′-ACACTCTTTCCCTACACGACGCTCTTCCGATCT(N/NN/NNN)TCTTGTGGAAAGGACGAAACAC-3′; reverse, 5′-CAAGCAGAAGACGGCATACGAGATXXXXXXGTGACTGGAGTTCAGACGTGTGCTCTTCCGATCT-3′; and XXXXXX-index.

Random nucleotides (NNN, NN, and N) were added to introduce sequence diversity, enhancing variability over the constant sequence for sequencing purposes. The DNA product was then purified with a 0.8:1 volumetric ratio of AMPure XP beads (Beckman Coulter A63881), and the whole volume was resuspended in 20 µL of PCR mastermix (KAPA HiFi ReadyMix) using the following primers: forward, 5′-AATGATACGGCGACCACCGAGATCTACACTCTTTCCCTACACGACGCTCTTCCGATCT-3′; reverse, 5′-CAAGCAGAAGACGGCATACGAGAT-3′.

PCR products were purified using a 0.8:1 volumetric ratio of AMPure XP beads, pooled, and sequenced on a single-end Illumina MiSeq run with 58 cycles (58 bp amplicon length) and an eight-cycle index.

### Single-cell sequencing

A single-cell suspension was loaded into the 10x Chromium device, and libraries were prepared using the Chromium Single Cell 3′ Library and gel bead kit v2 (10x Genomics PN-120237), following the manufacturer's instructions. The following samples were each loaded into a lane of the 10x Chromium control chip: day 4, day 10, *pZscan4c*-CreERT2 cells, gastruloids 1, gastruloids 2, and gastruloids 3.

Amplicon gRNA PCRs (KAPA HiFi ReadyMix) were performed for each of the 10x cDNA samples using 1 µL of the cDNA and the following primers: forward, 5′-ACACTCTTTCCCTACACGACGCTCTTCCGATCT(N/NN/NNN)TCTTGTGGAAAGGACGAAACAC-3′; reverse, 5′-AATGATACGGCGACCACCGAGATCTACACTCTTTCCCTACACGACGCTCTTCCGATCT-3′.

As before, random nucleotides (NNN, NN, and N) were added to introduce sequence diversity, enhancing variability over the constant sequence for sequencing purposes. PCR products were purified using a 0.8:1 volumetric ratio of AMPure XP beads, and the whole volume was loaded into a second nested PCR (KAPA HiFi ReadyMix) with the following primers: forward, 5′-CAAGCAGAAGACGGCATACGAGATXXXXXXXXGTGACTGGAGTTCAGACGTGTGCTCTTCCGATCT-3′; reverse, 5′-AATGATACGGCGACCACCGAGATCTACACTCTTTCCCTACACGACGCTCTTCCGATCT-3′; and XXXXXX- index.

After purification using AMPure XP beads, the enriched gRNA libraries were sequenced together with the transcriptome libraries on the Illumina NovaSeq platform.

### Bulk barcode analysis

A total of 161 samples were processed for bulk barcoding (see Supplemental Table S5). After sample demultiplexing, reads that did not contain both the flanking regions on the U6 promoter and the spacer regions on either side of the cassette were discarded (“AACAC” and “TTAGAG,” respectively). Barcodes with mean Illumina Phred scores below 28 were also removed. Additionally, samples with fewer than 200 reads were excluded. After this quality control, a total of 151 samples were processed for further analysis.

The percentage of original sequences was determined by comparing the spacer regions to the original spacer sequences. To assess divergence, each barcode was aligned to the reference spacer using the *pairwiseAlignment* function from the Biostrings R package (https://bioconductor.org/packages/release/bioc/html/Biostrings.html). The alignment was performed using a global–local approach with a substitution matrix that assigned a score of –1 for mismatches and +2 for matches, as well as a gap opening and extension penalties set to five. The divergence score for each sample was calculated as the average alignment score across its individual barcodes. Finally, divergence scores were normalized across all samples to ensure comparability. Aligned sequences were further analyzed to compute nucleotide frequencies per position.

### Single-cell transcriptome quality control and processing

All 10x scRNA-seq data were processed using the 10x Genomics Cell Ranger pipeline with the mm10 genome build, including the GFP sequence. For quality control, cells were discarded if they had fewer than 3000 detected genes and/or >7.5% of UMI reads originating from mitochondrial genes (Supplemental Fig. S16). Raw counts for each cell were normalized by dividing the counts by their size factors, and the resulting log-normalized counts were used for further analysis.

Broad clustering analysis and t-SNE plot generation were performed using Seurat (Satija et al. 2015). Linear dimensional reduction was executed via principal component analysis (PCA) using the Seurat function RunPCA, with highly variable genes identified by the Seurat function FindVariableGenes as inputs. Fifteen principal components were utilized to generate clusters and t-SNE plots using the Louvain algorithm implemented in the Seurat function FindClusters.

### Gastruloid cell type assignment

Cell types were assigned by aligning RNA expression profiles to a reference atlas as previously described (Argelaguet et al. 2019; Rosen et al. 2022). Briefly, count matrices from both data sets were combined and normalized. Highly variable genes were identified and used for PCA. Batch correction was performed to eliminate technical differences between the query and atlas cells. Using the integrated data, a *k*-nearest neighbors (kNN) graph was constructed. Each query cell's type was assigned by determining the most common cell type among the 30 nearest neighbors in the atlas, using a Dirichlet distribution for majority voting.

### Single-cell barcode library processing

FASTQ single-cell barcode libraries were processed to match cell IDs from the scRNA-seq transcriptomes. Reads lacking the specified flanking regions on the U6 promoter and spacer regions (“AACAC” and “TTAGAG,” respectively) or those with an Illumina Phred score of less than 28 were discarded (Supplemental Fig. S17). Furthermore, for each UMI with at least three barcode reads, a consensus barcode sequence was determined by selecting the sequence present in >50% of the retained UMIs. Divergence scores were calculated as in bulk analysis by performing a pairwise alignment of each barcode with the gRNA spacer, using the pairwiseAlignment function from the Biostrings package (https://bioconductor.org/packages/release/bioc/html/Biostrings.html). The divergence score for each cell was calculated as the average alignment score across its individual barcodes. Finally, divergence scores were normalized across all cells to ensure comparability.

### Stochastic simulation model

A stochastic simulation model was developed to describe 2C-like and pluripotent state dynamics. Each cell was represented as a binary particle (2C-like = 1, pluripotent = 0), with transitions governed by rate parameters *a* (2C-like → pluripotent) and *b* (pluripotent → 2C-like). At each time step, transition probabilities were calculated as

$$p_{\mathrm{down}}=1-e^{-a\;dt},p_{\mathrm{up}}=1-e^{-b\;dt},$$

where *p*_down_ and *p*_up_ correspond to the probabilities of exiting and entering the 2C-like state, respectively.

Simulations were run for N = 1000 cells over 12 days, with 1% initially 2C-like. Cells were updated independently, and the full state history was recorded. Parameter values were optimized to recapitulate experimental data by minimizing a loss function capturing deviations between simulated and observed metrics. Differential evolution (SciPy) was used to fit parameters (*a*, *b*), with repeated simulations to evaluate error (Virtanen et al. 2020). The best-fit parameters were then used to generate full temporal dynamics (N = 2000) for totipotent fraction, GFP+ accumulation, and re-entry behavior.

### Random forest model

The random forest model was employed to evaluate whether barcode editing over time could predict barcode duration in single cells. Single-cell data sets were used from days 0, 4, 6, and 10, whereby the mean barcode length, minimum barcode length, maximum barcode length, mean divergence, maximum divergence, and mean number of indels were incorporated as features. For each data set, 30% of the data was set aside for testing, whereas the remaining 70% was used for training. To address class imbalance, upsampling was applied to the training data using the upSample() function from the caret package (version 7.0-1). The randomForest function from the randomForest package (version *4.7-1.1*) was used, with “day” as the response variable and the selected features as predictors. Model performance on the test set was evaluated using classification accuracy and the F1 score, the latter defined as the harmonic mean of precision and recall to account for class imbalance.

## Data access

Sequencing data generated in this study have been submitted to the NCBI Gene Expression Omnibus (GEO; https://www.ncbi.nlm.nih.gov/geo/) under accession numbers GSE280613 and GSE280614. The scripts, processed data, and metadata are accessible on the GitHub repository (https://github.com/socyol/scDynaBar.git) and in the Supplemental Code.

## Supplemental Material

Supplement 1

Supplement 2
