# Supplementary material for: Using CRISPR barcoding as a molecular clock to capture dynamic processes at single-cell resolution
Source: Genome Res. 2026 May;36(5):1005–15. doi: 10.1101/gr.280915.125 (PMC13445707; doi:10.1101/gr.280915.125)
Supplement: Supplementary file 1 [file Supplemental_Figures.pdf]

GAGGGCCTATTTCATGATTCCTTCATATTTGCATATACGATACAAGGCTGTTAGAGAGATAATTGG
AATTAATTTGACTGTAAACACAAAGATATTAGTACAAAATACGTGACGTAGAAAGTAATAATTTCAT
TGGGTAGTTTGCAGTTTTAAAATTATGTTTTAAAATGGACTATCATATGCTTACCGTAACTTGAAAG
TATTTTGATTTCTTGGCTTTATATATCTTGTGGAAAGGACAAACACCGNNNNNNNNNNNNNNNNN
NNNGGGTTAGAGCTAGAAATAGCAAGTTAACCTAAGGCTAGTCCGTTATCAACTTGAAGAAAGTG
GCACCGAGTCGGTGCTTTTTTT

U6 promoter   TSS   Spacer   PAM   Scaffold   U6 terminator

ID	Spacer
g1	GTCCCCTCCACCCCACAGTG
g2	GTACACCCTCAAGCAGTGTG
g3	GTATGCGGATGCAATCTCCG
g4	GTCCCCTCCACCCCACTCTG
g5	CCTCACCTTCCTCACACCGC
g6	GTAGAGTCCCCTCCACCCCACAGTG
g7	ATCCACCTCACCTTCCTCACACCGC

Supplemental Fig. 1: The sequences of the gRNAs constructs used in this study.

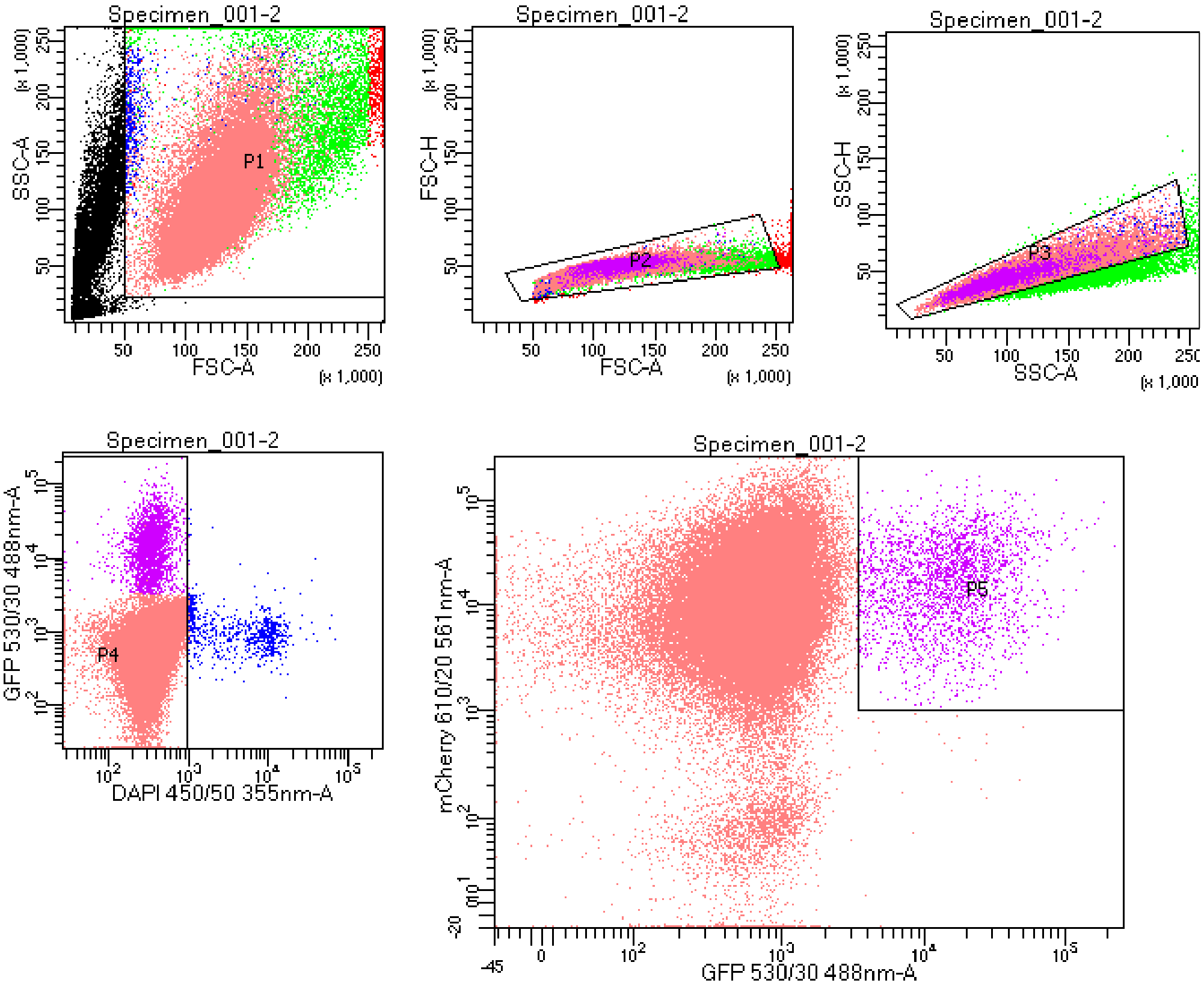

Supplemental Fig. 2: Cas9-activated cells were isolated by FACS by first gating on granularity and size (P1 gate), excluding doublets (P2 and P3 gates), selecting live cells (P4 gate 81.9%), and identifying mCherry (barcoding cassette) and GFP (Cas9) double-positive cells (P5 gate 5.4%).

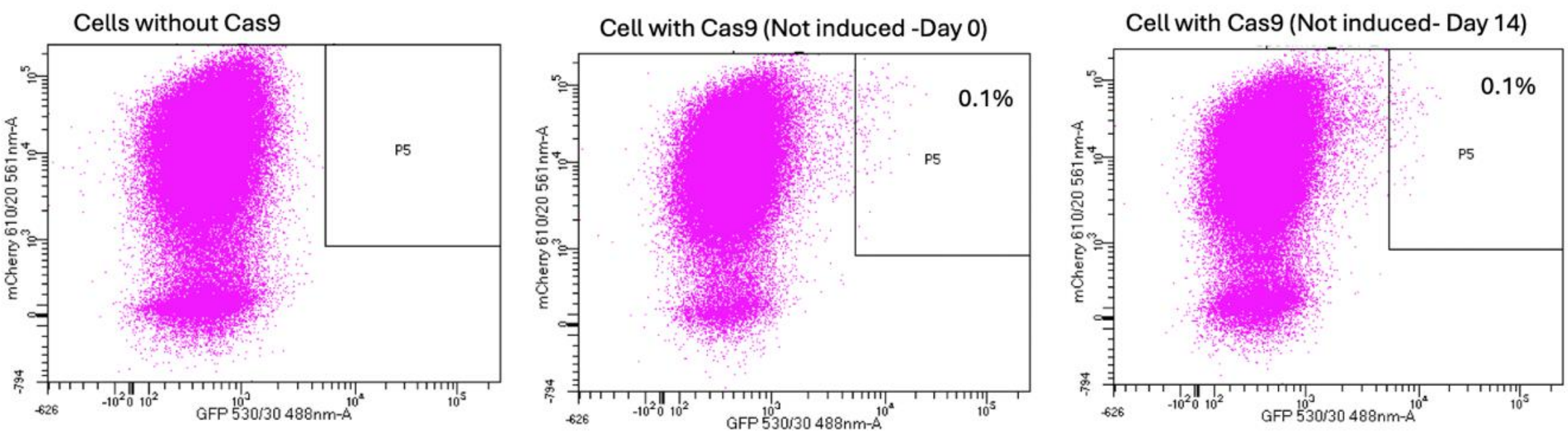

Supplemental Fig. 3: FACS analysis of three conditions: (left) cells containing the gRNA cassette without Cas9, (middle) cells with the gRNA cassette and uninduced Cas9, and (right) cells with the gRNA cassette and uninduced Cas9 after 14 days of culture. The scatter plot represents the distribution of cells based on mCherry expression (barcoding cassette, Y-axis) and Cas9-GFP expression (X-axis).

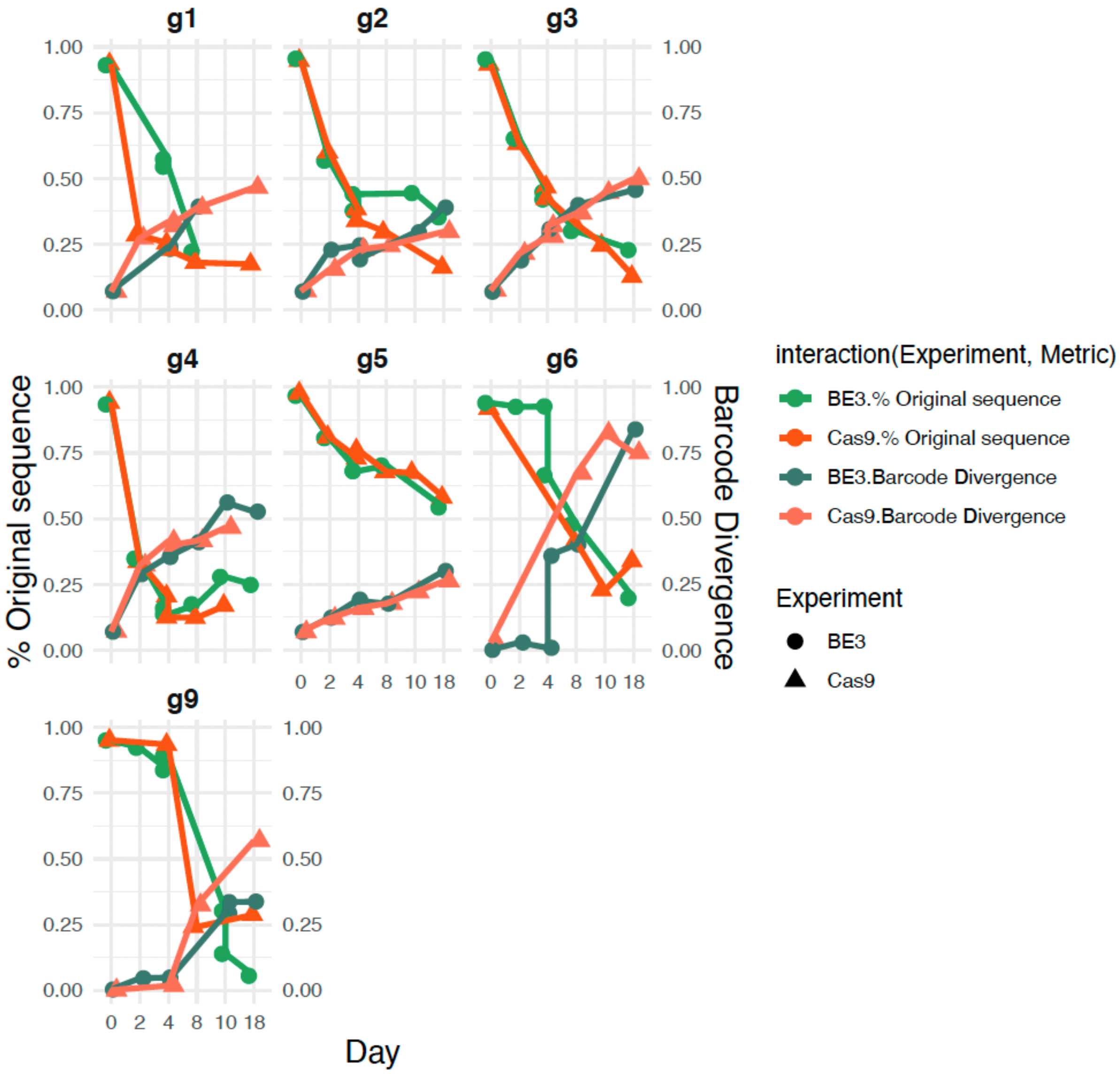

Supplemental Fig. 4: Proportion of original barcodes (left y-axis) together with barcode divergence from the original barcode sequence (right y-axis) over time (x-axis). Each point represents the mean value for one condition for a given combination of gRNA, system, and day (Cas9: n = 40; BE3: n = 42).

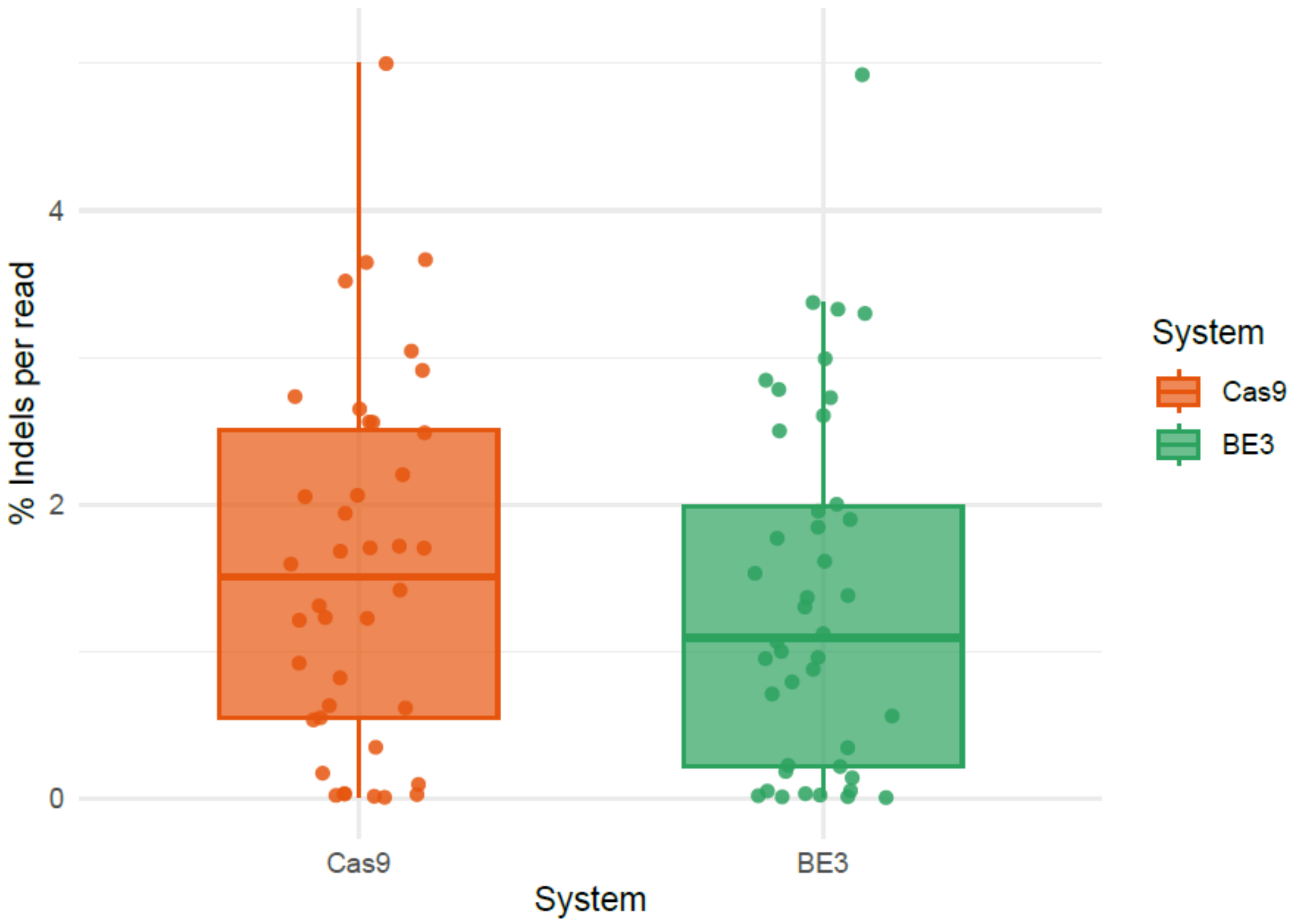

Supplemental Fig. 5: Average of indels per read grouped by system (BE3 (n=42) and canonical Cas9 (n=40)). (Mann-Whitney-Wilcoxon test; p = 0.45). The boxes represent the interquartile range (IQR), with the horizontal line inside each box indicating the median.

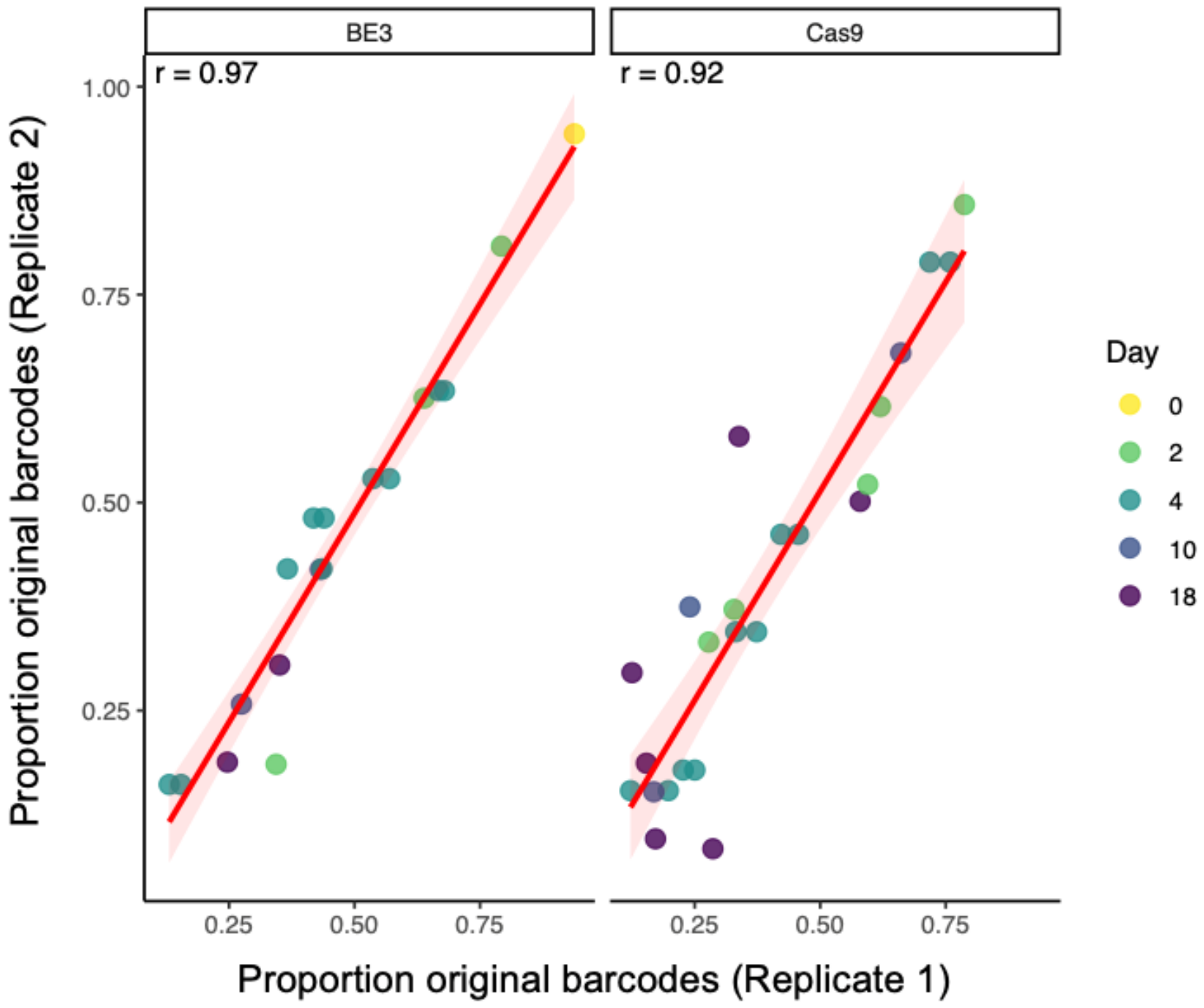

Supplemental Fig. 6: Scatter plots of original barcode frequencies in independent biological replicates, shown in separate panels by type of experiment. Replicate 1 is shown on the x-axis and replicate 2 on the y-axis. Points are colored by day. The Pearson correlation coefficient between replicates is shown in each panel.

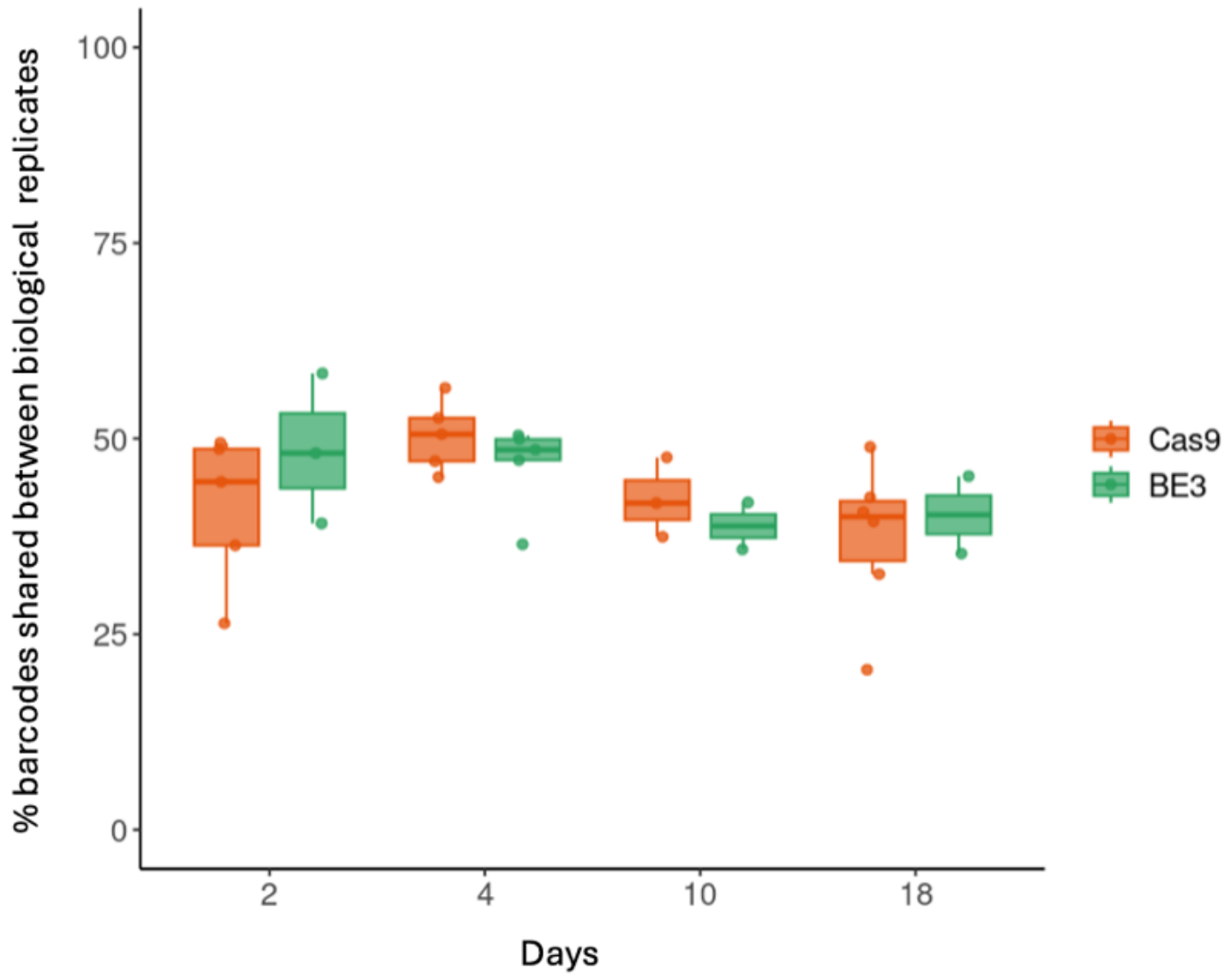

Supplemental Fig. 7: Boxplot showing the percentage of common barcodes shared between biological replicates for each time point and experiment (Cas9: n =50; BE3: n= 36). Boxplots indicate the median and interquartile range (IQR).

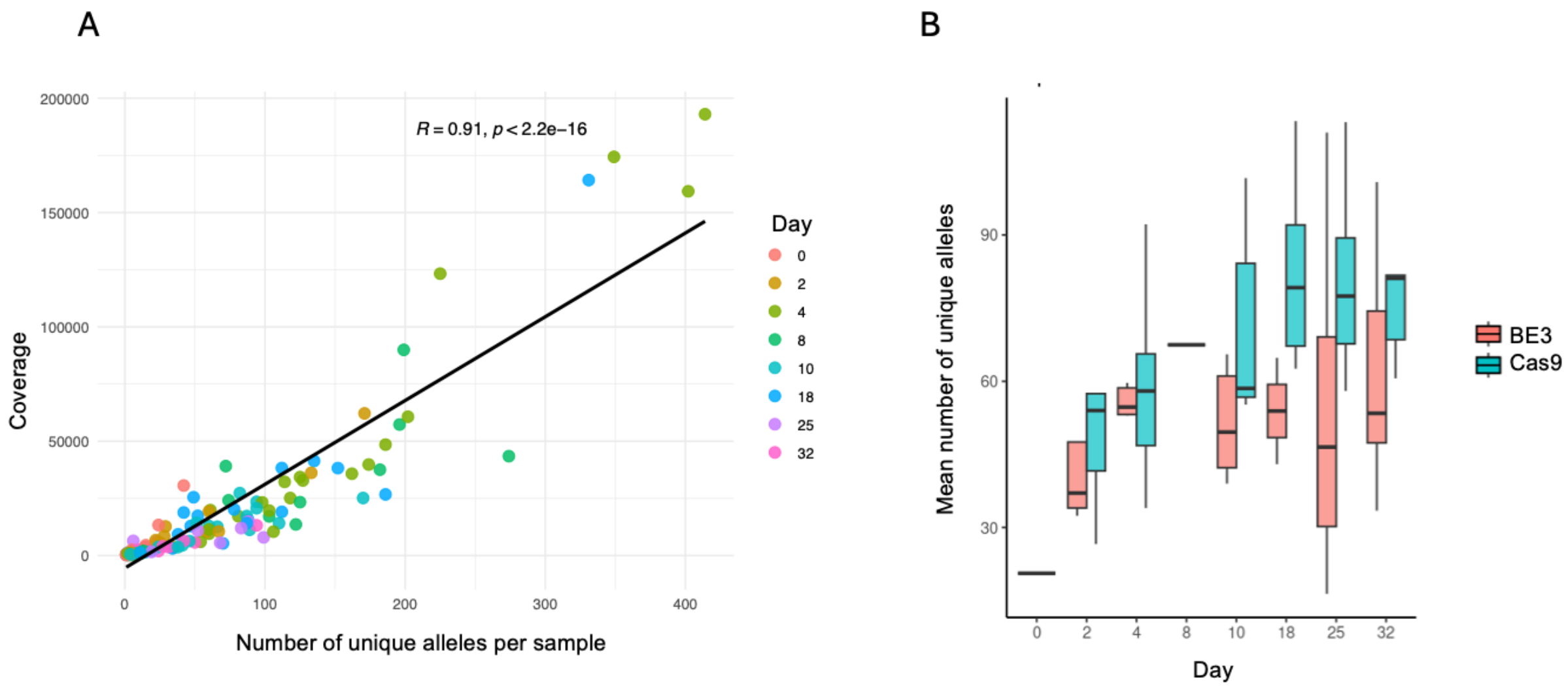

Supplemental Fig. 8: A. Scatter plot showing the relationship between sequencing coverage (total reads per sample) and the number of unique barcode sequences detected. Each point represents an individual sample coloured by day. **B.** Boxplots showing the number of unique barcode sequences per sample after bootstrapping at a standardized sequencing depth (5,000 reads). For each sample, subsampling was repeated 100 times. Boxplots represent the distribution of mean values per sample, with the center line indicating the median and boxes showing the interquartile range. Samples are grouped by experimental day (x-axis) and system (colour) (Cas9 n=39; BE3 n=29).

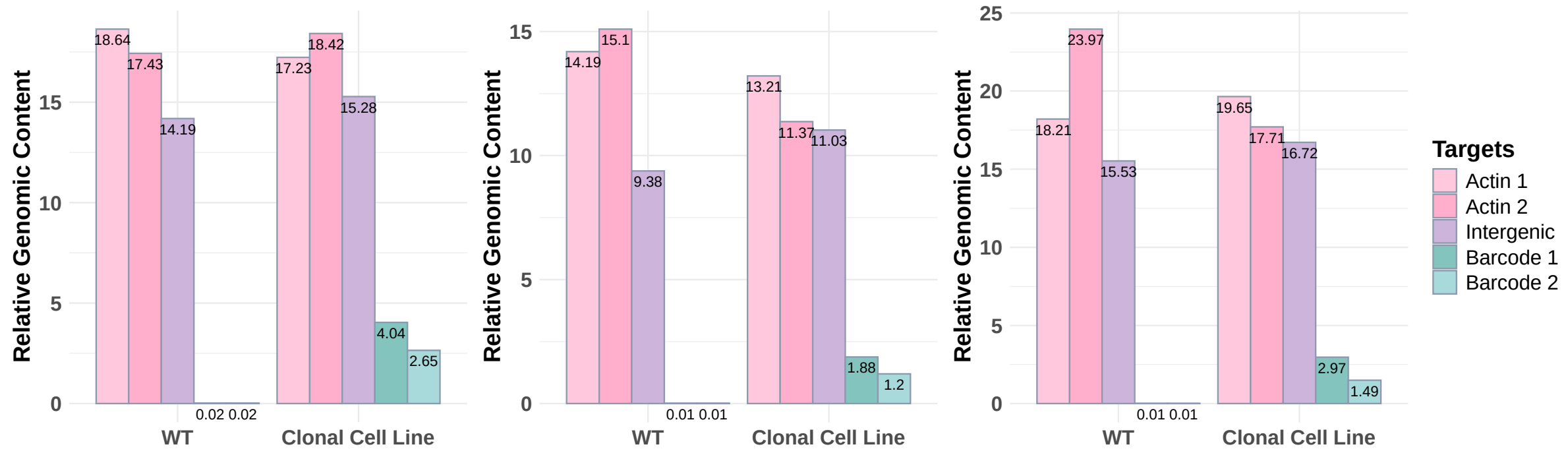

Supplemental Fig. 9. Relative genomic DNA content across five samples: Actin 1, Actin 2, intergenic region, Barcode 1, and Barcode 2. The actin locus and the barcoding cassette were amplified using two distinct primer sets (Actin 1 and Actin 2, Barcode 1 and Barcode 2). DNA content was normalized to the GAPDH locus, amplified with both primer sets. All results were performed in triplicate. The three panels represent different DNA concentrations: 5 ng (left), 10 ng (middle), and 20 ng (right). The higher signal for the actin and GAPDH loci compared to the barcode cassette suggests that the barcode cassette is present at a single-copy (1N) locus, while the endogenous loci are 2N, and may also reflect differences in PCR amplification efficiency between loci, likely due to sequence context.

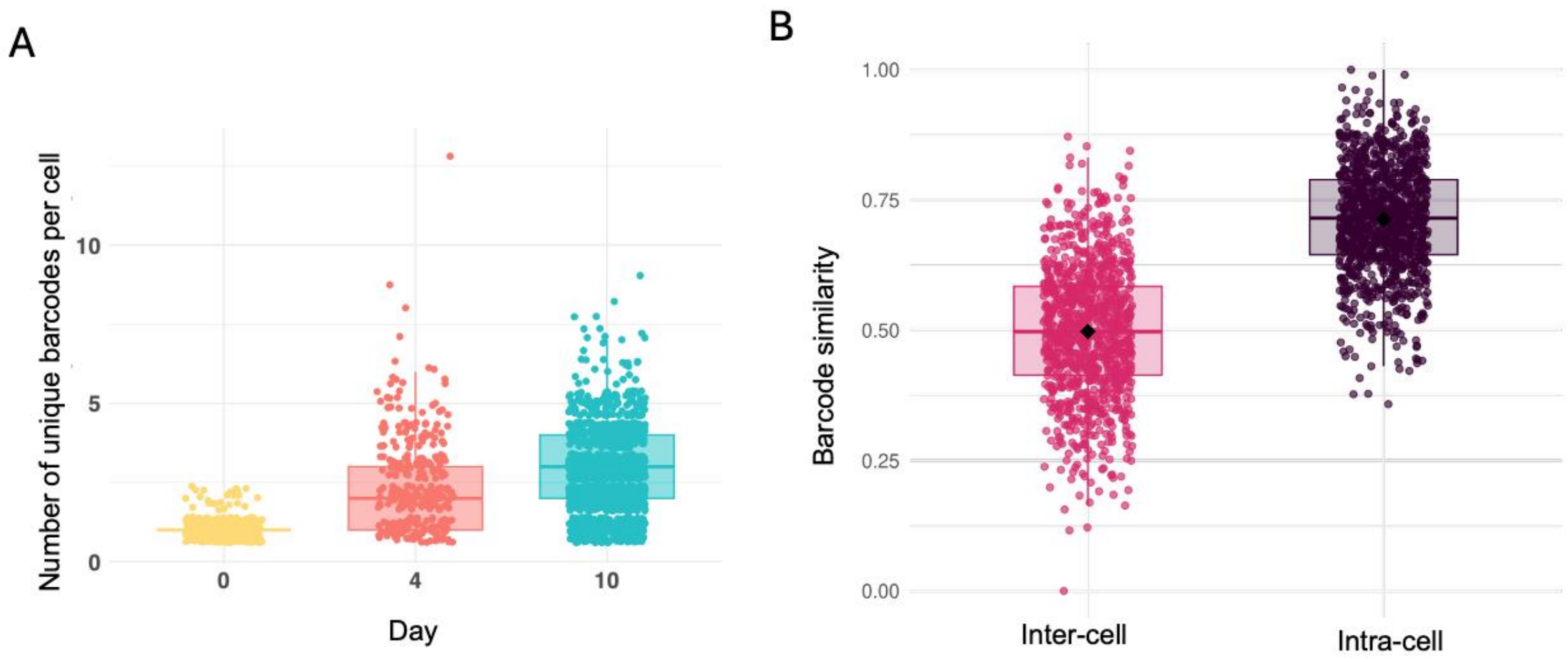

Supplemental Fig. 10. A. Number of unique barcode sequences per cell at days 0 (n=495), 4 (n=626) and 10 (n=1117). **B.** Boxplots of barcode sequence similarity scores for intra-cell (mean similarity among barcodes within a cell) and inter-cell (mean similarity among barcodes from different cells) ($p < 2.2 \times 10^{-16}$, Wilcoxon rank-sum test). In 1,000 bootstrap iterations, 15 cells were randomly sampled and mean similarity scores calculated. Each dot represents one iteration; boxes show the interquartile range (IQR) with the horizontal line inside each box indicating the median.

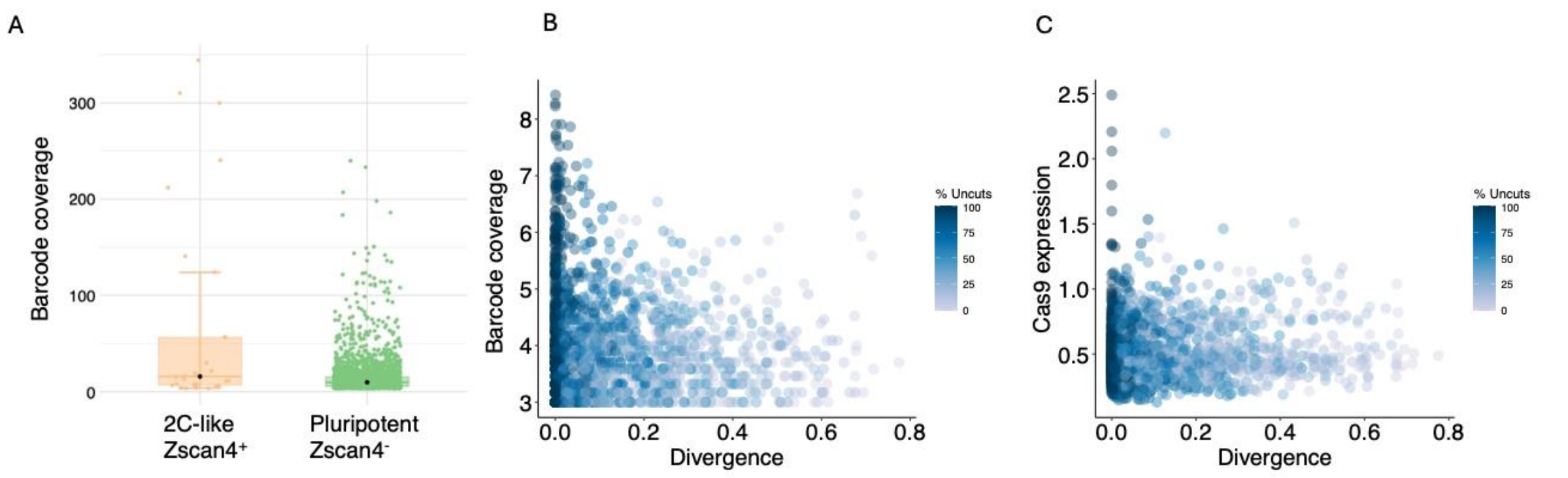

Supplemental Fig. 11. A. Boxplot showing the barcoding cassette coverage in 2C-like cells and pluripotent cells. The y-axis represents the number of reads**.** Coverage was calculated as the sum of edited and non-edited barcodes (median coverage Zscan4+: 16; median coverage Zscan4-: 10, Wilcoxon P-value = 0.037). **B.** Scatterplot showing the relationship between barcode divergence and barcode coverage (Pearson's r = -0.12, P-value = 1.0×10^{-6}) **C**. Scatterplot showing the relationship between barcode divergence and Cas9 expression. Each dot represents a single cell; colour indicates the percentage of original (uncut) sequences (Pearson's r = 0.05, P-value = 0.041).

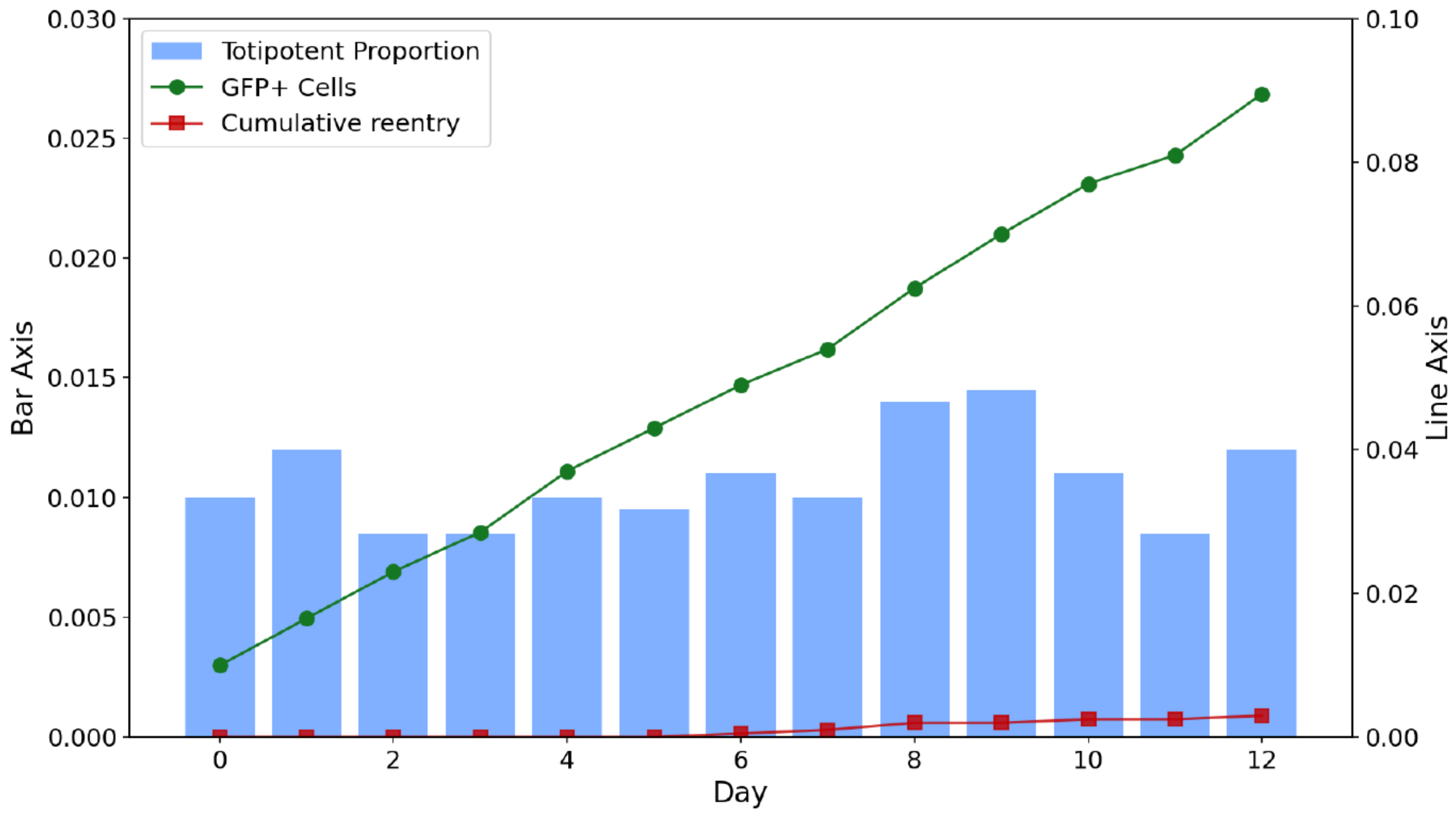

Supplemental Fig. 12. Boxplot Proportion of cells in the 2C-like state at each time point (blue bars). Cumulative fraction of cells that have ever entered the 2C-like state is shown with red circles, and the fraction of cells that re-entered the 2C-like state after at least one exit is shown with red squares.

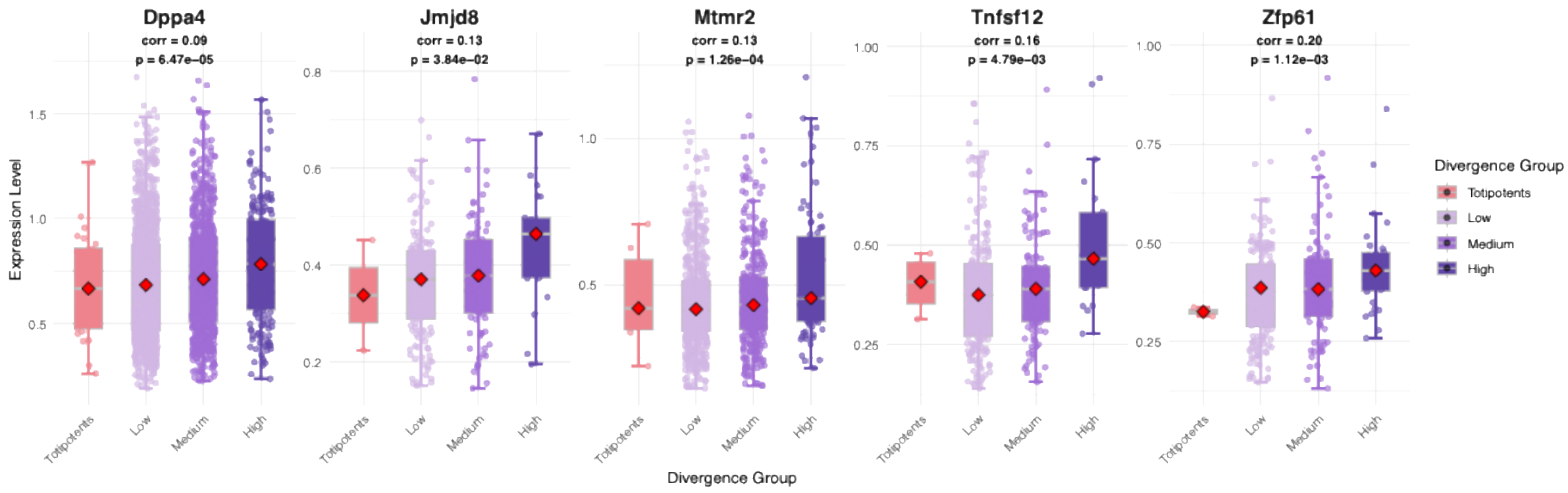

Supplemental Fig. 13. Examples of genes whose expression levels are correlated with the barcode divergence score. The boxplot shows the log2-transformed expression levels, grouped by levels of divergence: Low, Medium, and High. Pearson correlation and p-value are displayed at the top.

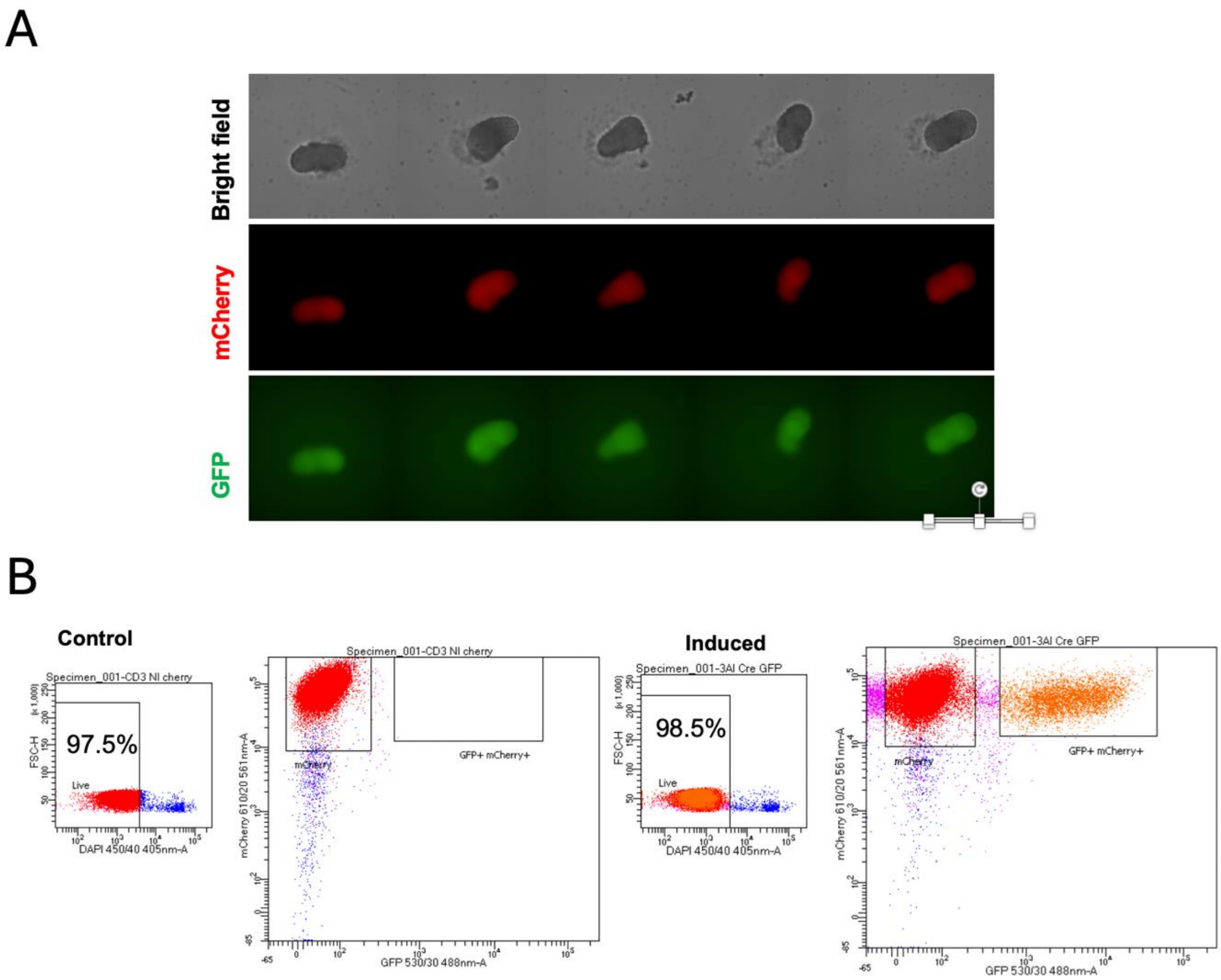

Supplemental Fig. 14: **A**. Representative bright field, mCherry, and GFP images of gastruloids after six days of barcoding (corresponding to Day 4 of differentiation). **B**. FACS analysis of gastruloid samples. Left: control gastruloids (without barcoding); Right: induced gastruloids (with barcoding). DAPI was used to assess cell viability (x-axis), with the gated percentages indicating viable cells. The adjacent plot displays the proportion of mCherry-positive cells (y-axis) alongside GFP expression (right axis).

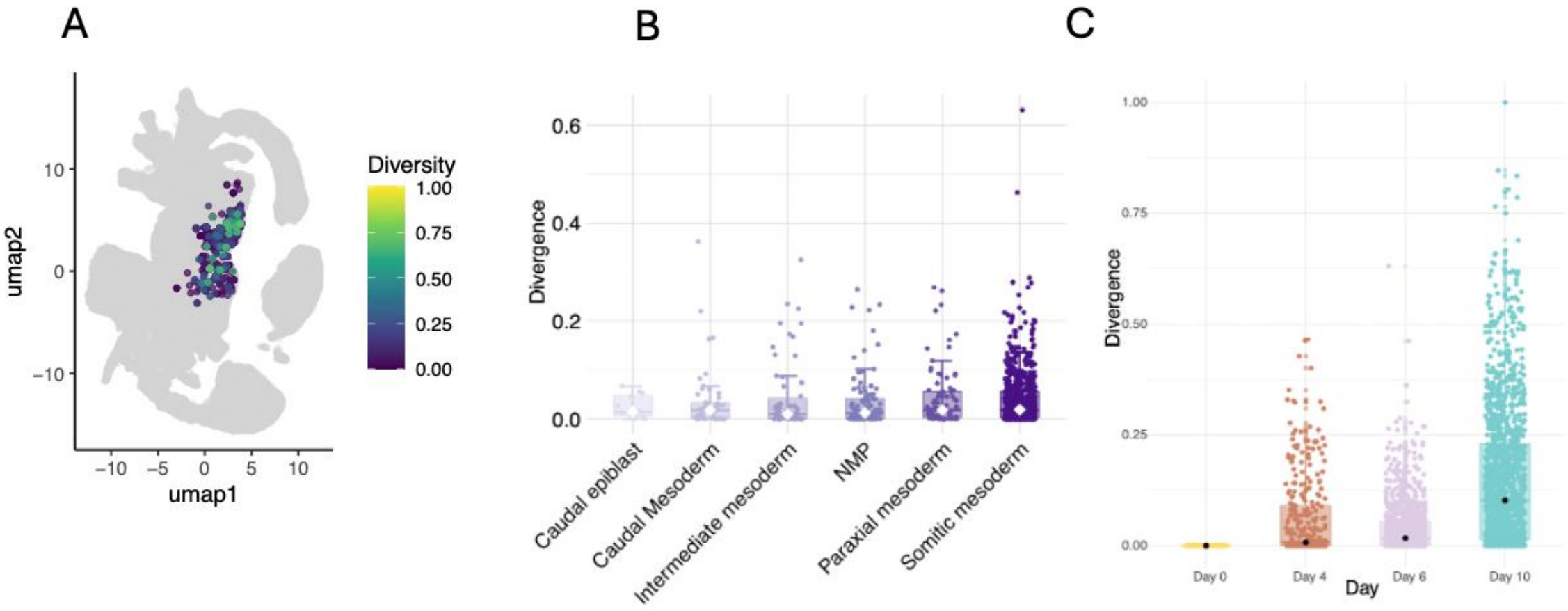

Supplemental Fig. 15: **A.** UMAP projection of a reference atlas of mouse embryos (E6.5 and E8.5). Gastruloid cells are overlaid and coloured by barcode divergence. **B.** Boxplot showing the barcode divergence score for each cell grouped by cell type (Kruskal-Wallis, p-value = 0.319). **C.** Boxplot showing the barcode divergence score across different time points in single cells (Jonckheere-Terpstra test, $p = 2 \times 10^{-16}$,1000 permutations).

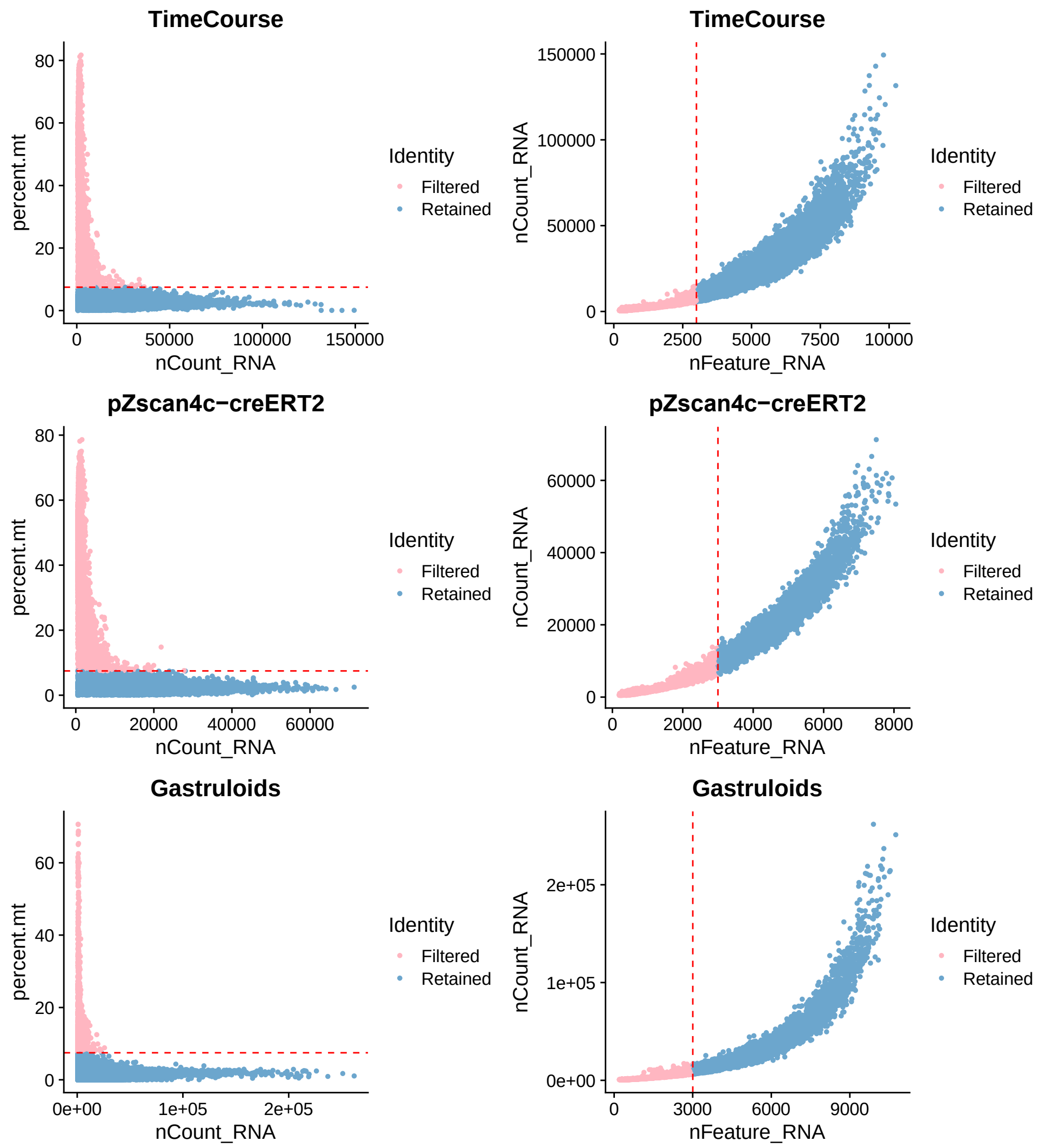

Supplemental Fig. 16: Quality Control of Single-Cell RNA-seq Data. The plots display the number of genes (nFeature_RNA) and the percentage of mitochondrial reads (percent.mt) versus total expression counts (nCount_RNA) per cell for each experiment. Cells with more than 3,000 genes and/or less than 7.5% of UMI reads originating from mitochondrial genes were included in the study.

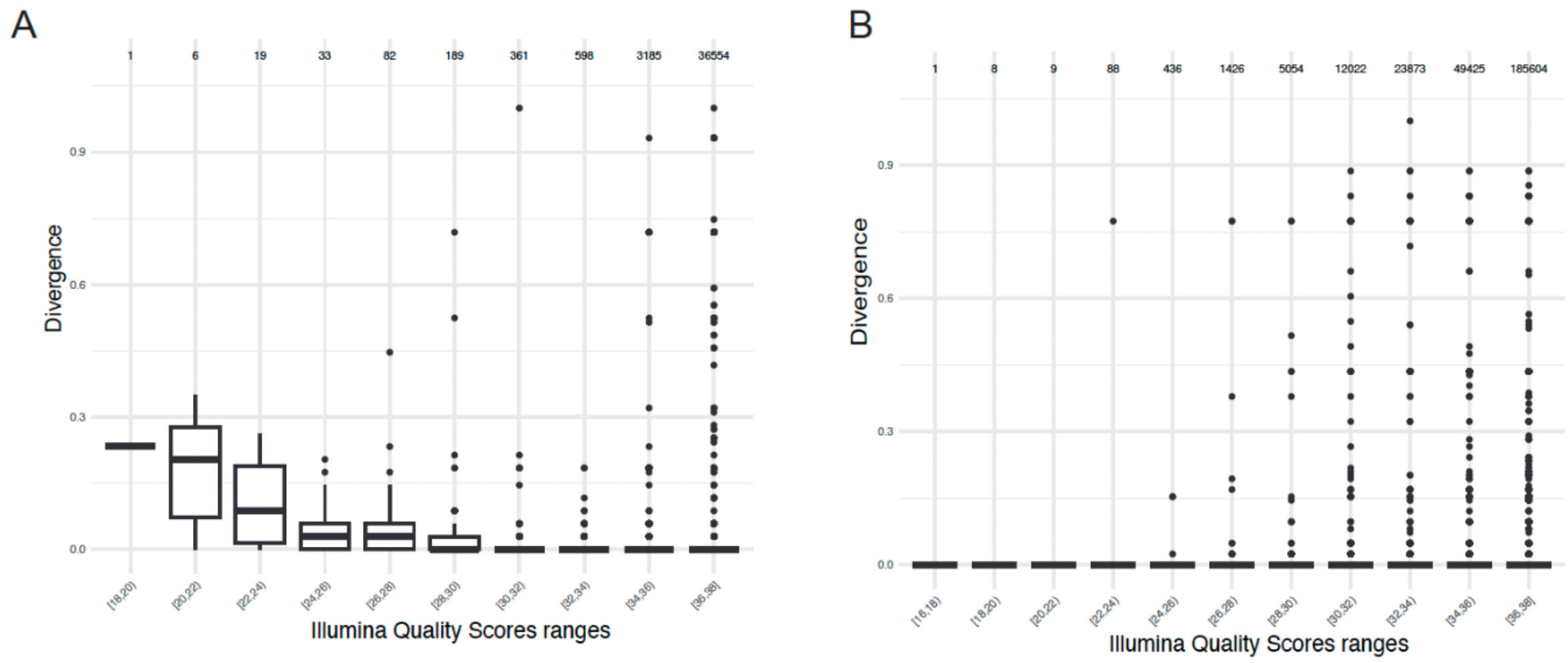

Supplemental Fig. 17: Barcode read quality control. Barcode divergence from the original barcode sequence versus mean Illumina sequencing quality score for the single-cell barcode library of Day 0 (A) and the control group of the Zscan4 experiment (B). The number displayed above each boxplot indicates the number of observations.
